# Supplementary material for: Super-resolution techniques to simulate electronic spectra of large molecular systems
Source: Nat Commun. 2024 Sep 12;15:8001. doi: 10.1038/s41467-024-52368-5 (PMC11393058; doi:10.1038/s41467-024-52368-5)
Supplement: Supplementary file 1 — supplementary_information [file 41467_2024_52368_MOESM1_ESM.pdf]

Supporting Information:
Super-resolution techniques to simulate electronic spectra of large molecular systems

Matthias Kick*[1], Ezra Alexander[1], Anton Beiersdorfer[2], and Troy Van Voorhis[1]

[1]Department of Chemistry, Massachusetts Institute of Technology, Cambridge, Massachusetts 02139, USA
[2]Technical University of Munich, Lichtenbergstrasse 4, Garching 85747, Germany

August 8, 2024

1 Algorithm: Details

We have already discussed the basic working principles of our algorithm in the main text. In this section of our supplementary information, we describe in detail how our algorithm deals with a challenging initial guess. Towards this purpose, we briefly reiterate the main steps of BYND.

We start with an initial set of frequencies obtained from the SMA. To begin, the amplitudes are adjusted using linear regression. The subsequent non-linear optimization of the SMA frequencies is the heart of BYND, which is realized as a greedy-algorithm.[1] Our algorithm starts with the frequencies which have the highest amplitude and optimizes their position by performing a line-search around the initial frequency with a search space of $\pm\Delta\omega$. If a minimum is found, the algorithm updates the old value with the newly found optimum frequency value and moves forward to the next frequency. The amplitude fitting procedure and the frequency optimization are then repeated until convergence is reached. The amplitude adjustment step causes frequencies which are too far away from their correct values to be filtered out by setting their amplitude to zero. This can be problematic in cases where the SMA provides a highly-inaccurate initial guess, namely when bright states are incorrectly set to zero amplitude due to their SMA frequencies being too far from the target value. This can be effectively prevented by setting a high initial value of $\Delta\omega$, between 0.3 - 0.8 a.u. ($\Delta\omega_{\text{init}}$). All following iterations can then use a smaller value of $\Delta\omega_{\text{def}} = 0.001$ a.u.. As an additional precaution, before we enter our frequency line-search procedure for the first time we modify each amplitude $A_k^{\lambda\mu}$ by randomly adding a value between zero and 10% of the highest amplitude. This ensures that every frequency will be considered in the calculation of the loss function. The entire procedure is described in Supplementary Algorithm 1. We have found that both a high $\Delta\omega_{\text{init}}$ and the random adjustment of the amplitudes in the first iteration are necessary to converge initial guesses where the frequencies are far from their final values. In Supplementary Figure 1 we compare the final spectrum of the $Cd_{38}Se_{38}$-ZnPc-32(NH_2CH_3) nanocrystal with the case where we do not broaden $\Delta\omega$ or add random amplitude on the first iteration. As one can clearly see, the algorithm fails to correctly locate the frequency at 5.4 eV without our modifications (Supplementary Figure 1b). Here the closest frequency in the SMA initial guess is the frequency at around 4.8 eV (Supplementary Figure 1a), but the default search grid is too small to correct its position. As a result, its amplitude is set to zero. With a large enough $\Delta\omega_{\text{init}}$ and random adjustment of the amplitudes in the first iteration we can effectively prevent such cases and obtain the correct excitation spectrum (Supplementary Figure 1c).

In addition, one can improve the overall convergence of the algorithm by using only dipole moments where the applied electric field pulse is parallel with the direction of the dipole operator for the first few iterations. All other signals should then be included for the remaining iterations. We find that the non-negative constraint helps to reduce the number of degrees of freedom in the amplitude fitting procedure and thus allows the algorithm to be more robust in the first few iterations. Sometimes and analogous to when using Fourier transform techniques, the electric field pulse at the beginning can cause unwanted spectral artifacts. One may approach this by cutting the signal accordingly or by applying some window function techniques.

*mkick@mit.edu

SupplementaryAlgorithm 1 Line-search

initial guess for ω_k from SMA
while not converged **do**
  $A_k^{\lambda\mu} \leftarrow \min \frac{1}{n} \sum_i ||y_i^{\lambda\mu} - f_{\lambda\mu}^{\text{sparse}}(A_k^{\lambda\mu}, \omega_k, t_i)||_2^2 + \alpha ||f^{\text{sparse}}||_2^2$
  if iteration $= 1$ **then**
    $\Delta\omega \leftarrow \Delta\omega_{\text{init}}$
    randomly modify $A_k^{\lambda\mu}$
  else
    $\Delta\omega \leftarrow \Delta\omega_{\text{def}}$
  for $\omega_i \in \{\omega_1, ..., \omega_k\}$ **do**
    for $\omega \in \{\omega_i - \Delta\omega, ..., \omega_i, ..., \omega_i + \Delta\omega\}$ **do**
      $f_{\lambda\mu} \leftarrow -\sum_{k \neq i} A_k^{\lambda\mu} \sin(\omega_k t_i) + A_i^{\lambda\mu} \sin(\omega t_i)$
      Compute $L\left(A_k^{\lambda\mu}, A_i^{\lambda\mu}, \omega_k, \omega\right), \quad k \neq i$
    $\omega_i \leftarrow \min(L)$
    $A_k^{\lambda\mu} \leftarrow \min \frac{1}{n} \sum_i ||y_i^{\lambda\mu} - f_{\lambda\mu}^{\text{sparse}}(A_k^{\lambda\mu}, \omega_k, t_i)||_2^2 + \alpha ||f^{\text{sparse}}||_2^2$

2 Limitations regarding the SMA input frequencies

2.1 Too many frequencies

Our approach uses the SMA frequencies as an initial guess for the location of the narrow features. As SMA frequencies are highly approximate, they are error prone with shifts of up to 0.5 eV. Moreover, there are often more narrow SMA frequencies than there are exact narrow features. One particular reason for this, among others, is that the SMA breaks symmetry. We address this by limiting the number of initial guess frequencies that we will fit using a user-defined threshold, e.g. all frequencies for which the transition dipole moment is above a specified value. While useful, this empirical parameter adds a level of ambiguity to the way we obtain the initial guess. We will demonstrate that this is usually not a problem for our algorithm as our method is robust enough to deal with an initial guess which contains too many frequencies. For this purpose, we consider the $Cd_{38}Se_{38}$-ZnPc-32(NH_2CH_3) example system. This nanocrystal with a chromophore adsorbed has three distinct narrow features and thus serves as a clear example of the behavior of our algorithm when too many initial frequencies are selected.

In Supplementary Figures 2 and 3, we show how the final results for $Cd_{38}Se_{38}$-ZnPc-32(NH_2CH_3) depend on the chosen number of input frequencies. We choose a set of 4, 9, 11, and 22 frequencies and monitor how the frequencies and their amplitude change with each iteration of our algorithm, also displaying the final dipole spectrum in each case. We repeat this analysis for two different target signal lengths: 500 and 1,000 time steps.

In Supplementary Figure 2a we start with four input frequencies. The final dipole spectrum should have three narrow features, and in fact after only a few iterations our algorithm optimizes two of the input frequencies to have the same energy (around 2 eV). These two frequencies also show almost identical amplitude. The same observation holds true if we go to the case where we have nine initial guess frequencies (Supplementary Figure 2a). Our algorithm optimizes three frequencies (orange, green and red) to have the same value. These frequencies form the narrow feature at around 1.9 eV, and again our algorithm assigns to each of these three frequencies almost identical amplitudes. We would also like to highlight the behaviour of the brown and the blue frequencies, both of which drop to zero amplitude after iteration 50 and 60, respectively. This demonstrates another way in which our algorithm can deal with excess initial guess frequencies. Evidence of grouping excess frequencies together to one specific value or setting the amplitudes of excess frequencies to zero can still be seen when we extend to a larger number of initial guess frequencies (see Supplementary Figure 2c and 2d).

We observe the same behaviour even when we reduce the number of time steps to 500. Our spectra remains well reproduced when we employ 4, 9, and 11 initial frequencies (Supplementary Figure 3a, Supplementary Figure 2b and 2c), with excess frequencies set to an amplitude of zero or grouped together. However, with 22 input frequencies we observe that the algorithm erroneously predicts an additional high amplitude narrow feature with an excitation energy of 0.3 eV. While we saw a frequency with similar excitation energy when we had 1,000 time steps, there the algorithm was able to set the amplitude of that frequency to zero. Thus we conclude that 500 time steps does not provide us with enough data to allow the algorithm to filter out this frequency. However, as this artifact was not present with fewer initial guess excess frequencies, one will be able to identify and correct for this behaviour by simply reducing the number of initial guess frequencies and observing any changes in the dipole spectrum.

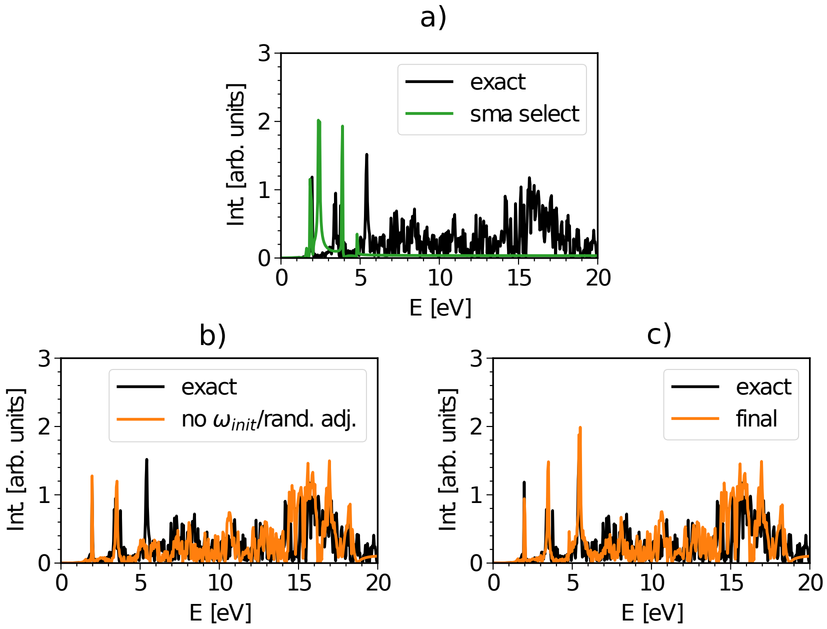

Supplementary Figure 1: In a) we show the absorption spectrum of the selected SMA frequencies only. b) shows the complete absorption spectrum without $\Delta\omega_{\text{init}}$ and random adjustment of the amplitudes . In c) we display the full absorption spectrum with $\Delta\omega_{\text{init}}$ and random adjustment of the amplitudes. The excitation at 4.8 eV is too far away for a search grid of 0.001 a.u. and the algorithm is not able to correctly locate the excitation at around 5.4 eV. With a large enough $\Delta\omega_{\text{init}}$ and random adjustment of the amplitudes in the first iteration, we can effectively prevent such cases. In order to achieve better comparability and for the sake of simplicity, the intensities of the SMA excitations have been reduced to two thirds of their original values.

A similar approach can be applied in the case where one selects too few initial guess frequencies. With too few initial guess frequencies certain narrow features will be missing from the final spectrum, but this can easily be tested by simply adding more initial guesses and checking to see if additional features appear. We also present in section 2.2 of our supplementary information a more systematic way to identify and quantify situations where not enough frequencies have been selected. In general, we suggest always performing multiple optimization runs with a range of different initial guess frequencies.

We conclude that our method is robust regarding the number of initial guess frequencies. It can deal with an initial guess where the number of frequencies greatly exceeds the number of target narrow features. As the computational cost of our method is minimal, one can easily perform several optimization runs with a different number of initial guess frequencies in order to identify spectral artifacts and situations where the number of initial guess frequencies is smaller than the actual number of narrow features. As a general rule of thumb, such issues become less prominent when one utilizes a longer real-time simulation.

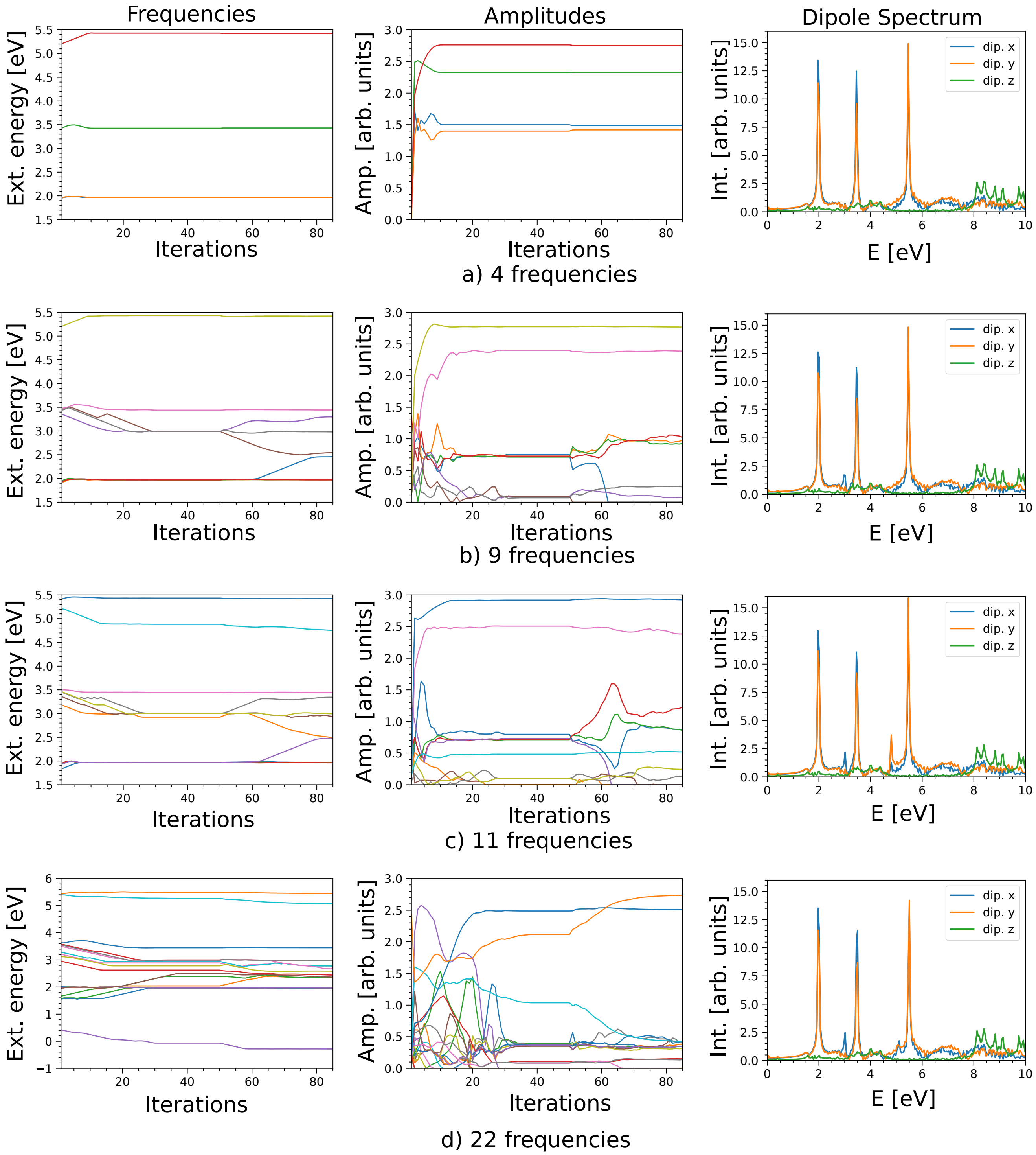

Supplementary Figure 2: Influence of the number of initial guess frequencies on the final result. a) Four selected initial frequencies, b) nine selected frequencies, c) 11 initial frequencies and d) 22 frequencies. On the left we show the excitation energies (frequencies) for each iteration. In the middle we show the amplitudes of each frequency at each iteration. For simplicity, we combine the amplitudes for the x-, y- and z-dipole to one value per frequency. Amplitudes and frequencies associated with each other have the same color. On the right we show the electronic dipole spectrum of the final result. Here, results have been obtained with 1,000 data points.

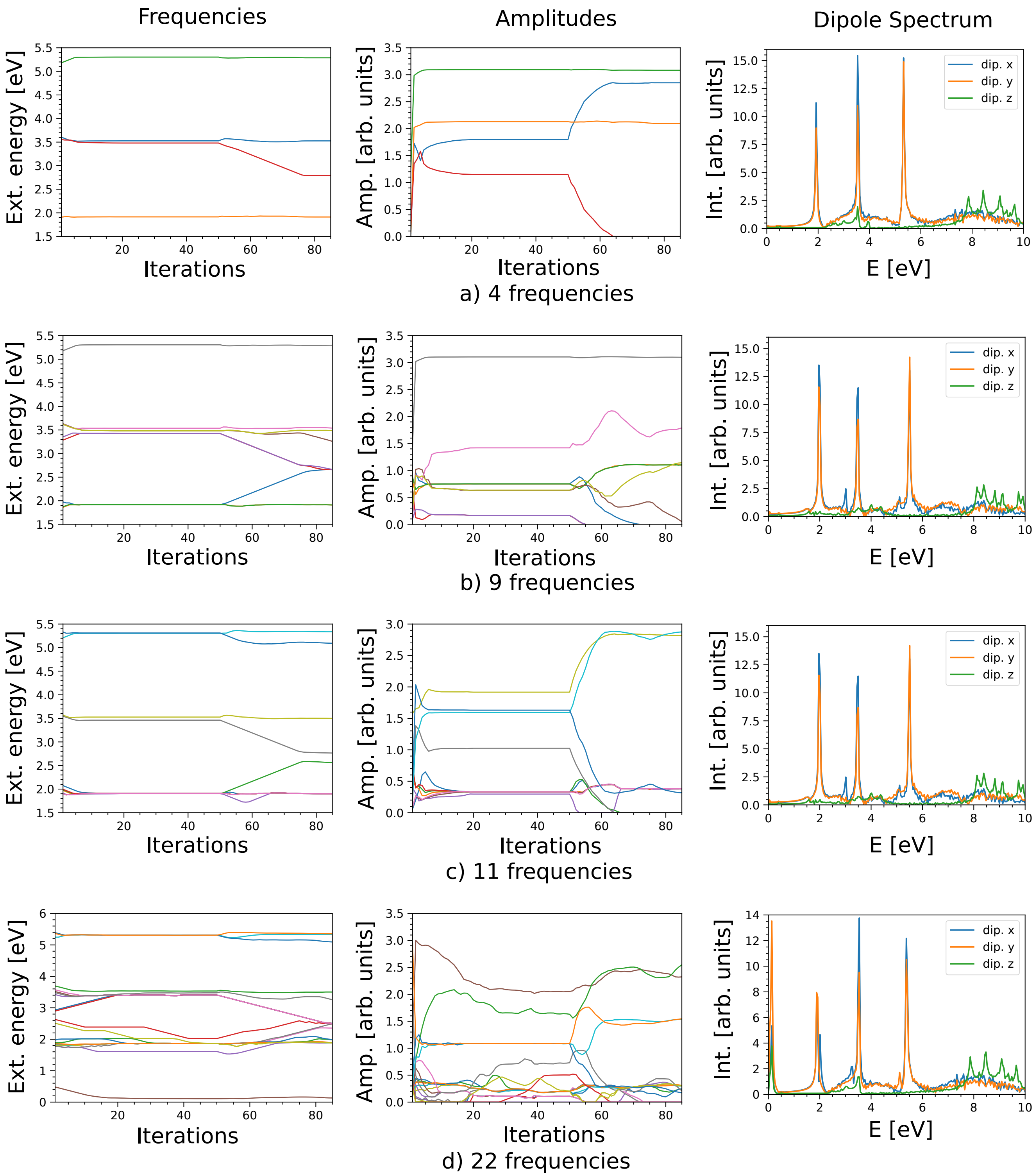

Supplementary Figure 3: Influence of the number of initial guess frequencies on the final result. a) Four selected initial frequencies, b) nine selected frequencies, c) 11 initial frequencies and d) 22 frequencies. On the left we show the excitation energies (frequencies) for each iteration. In the middle we show the amplitudes of each frequency at each iteration. For simplicity, we combine the amplitudes for the x-, y- and z-dipole to one value per frequency. Amplitudes and frequencies associated with each other have the same color. On the right we show the electronic dipole spectrum of the final result. Here, results have been obtained with 500 time steps.

2.2 Not enough frequencies or frequencies too far away

Another limitation associated with the SMA input is when an initial guess frequency is far away from its target position. As we show in Supplementary Figure 4, the butadiene molecule is an example of such a case. Here, the intense peak at about 5.4 eV appears at about 7.0 eV in the SMA spectrum. There are no other nearby frequencies in the SMA spectrum, as the next SMA frequency appears at around 11 eV. As a consequence, a simple linear prediction starting from the SMA frequencies fails. However, as we show in Supplementary Figure 4 BYND is able to find the correct peak positions as long as $\Delta\omega_{\text{init}}$ is large enough. For the intense peak in butadiene, this value is $\Delta\omega_{\text{init}} = 0.05$ a.u. (1.35 eV). In order to reproduce the spectral features above 10 eV, smaller $\Delta\omega_{\text{init}}$ are sufficient as the input SMA frequencies are much closer to the the final result.

An easy way to spot such situations is to calculate the coefficient of determination (R^2) between the target signal and the signal obtained from the narrow feature optimization with a range of $\Delta\omega_{\text{init}}$ values. In Supplementary Figure 5, we show R^2 values for each different dipole signal of butadiene at different values of $\Delta\omega_{\text{init}}$. For $\Delta\omega_{\text{init}}$ values below 0.05 a.u., we observe only moderate changes in R^2. In these cases, the aforementioned high-energy peaks that are well reproduced by the SMA have already been shifted to the correct positions. At $\Delta\omega_{\text{init}} = 0.05$ a.u., BYND is able to fit the first intense peak and the R^2 values improve significantly. Further increasing $\Delta\omega_{\text{init}}$ to 0.07 a.u. does not result in any further changes in R^2 and thus we reach convergence with respect to $\Delta\omega_{\text{init}}$. Thus, calculating R^2 values is a simple way to identify situations where our default $\Delta\omega_{\text{init}}$ value might be too small.

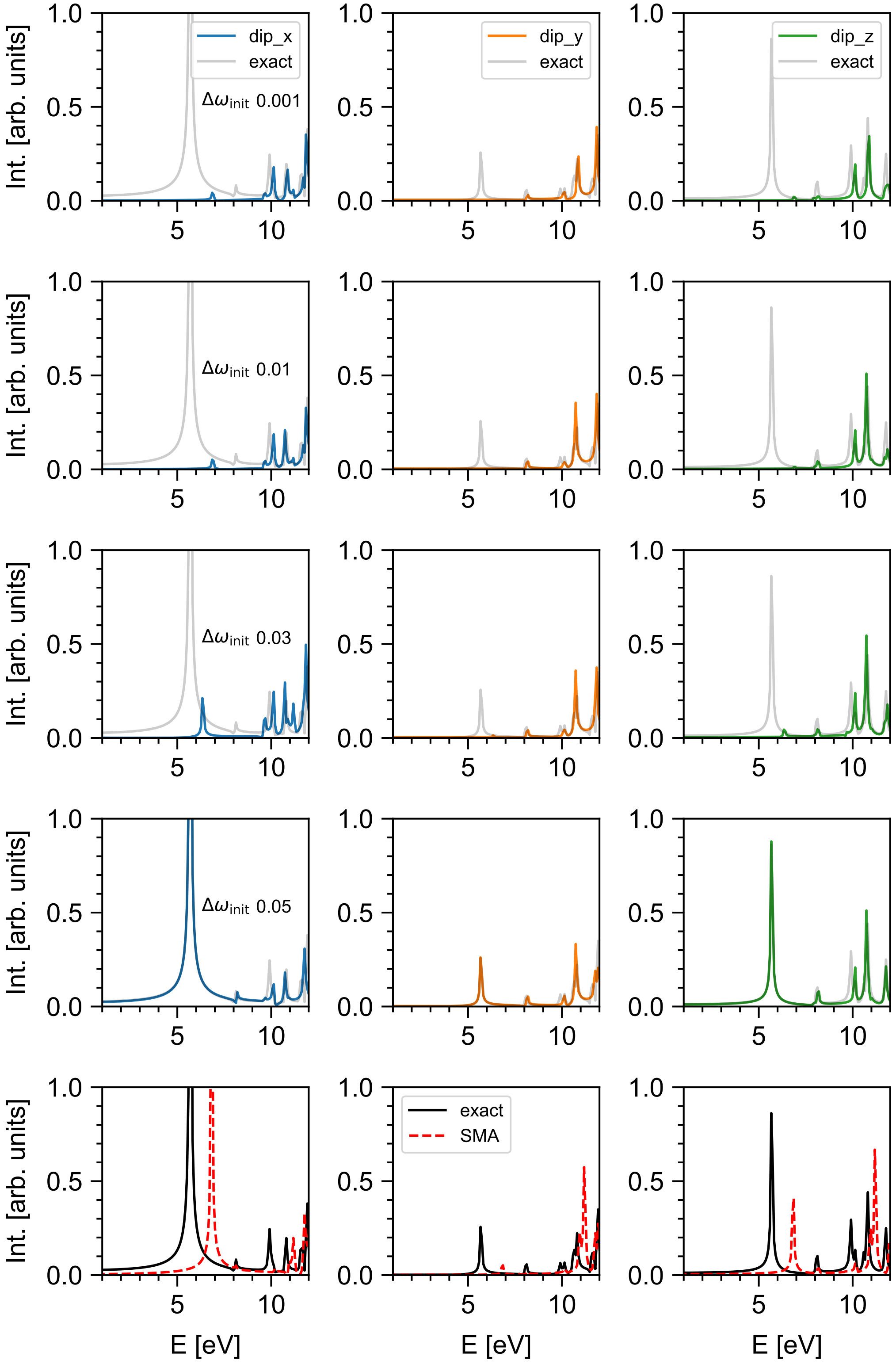

Supplementary Figure 4: Dipole signals of a butadiene molecule obtained from BYND using different $\Delta\omega_{\mathrm{init}}$. We only show the dipole signals where the initial electric field vector and observed dipole are parallel. We also show the exact result from a long-time simulation and the SMA spectrum for comparison.

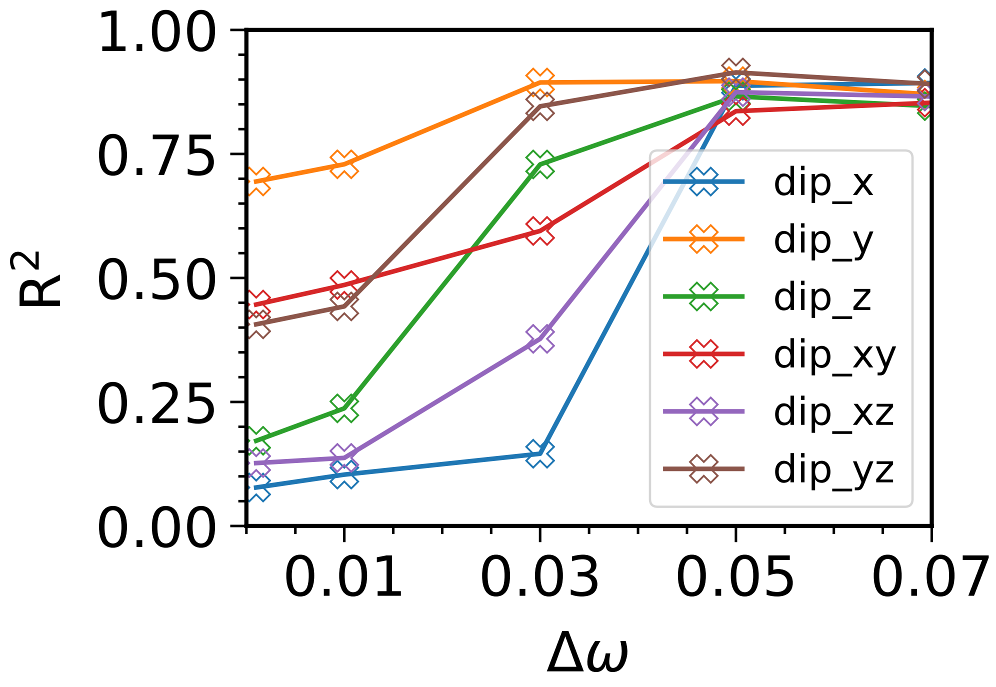

Supplementary Figure 5: Calculated R^2 values between the BYND narrow feature position and that of the target signal for butadiene. Results are shown for all dipole signals, including those where the initial electric field pulse vector and observed dipole are not parallel (xy, xz and yz).

Another limitation of our SMA initial guess is the case where not enough frequencies have been selected. In Section 2.1, we briefly discussed strategies to spot such a situation. Here, we will demonstrate such cases with a much more challenging example: a $Cd_{38}Se_{38}$-ZnPc-DPA-32(NH_2CH_3) nanocrystal. We will focus on the region at around 6.8 eV. In this region, we expect several distinct narrow features with similar frequencies. In order to demonstrate the effect of selecting too few frequencies, we vary the number of initial frequencies from 1 to 5 for this region. The selected frequencies for this region are given in Supplementary Table 1, and Supplementary Figure 6 shows the corresponding dipole spectra compared to the exact dipole spectrum from a long-time simulation. In Supplementary Figure 6a, only one frequency is selected and only one narrow feature is found (z-dipole). In b) a second frequency is present, and now an additional narrow feature (in the x-dipole) is found. We only find all three narrow features when we select three initial frequencies (6c)). There is no further change upon adding more initial frequencies, indicating that all narrow features are correctly reproduced at three initial frequencies.

In order to spot situations where too few frequencies have been selected, we again calculate the R^2 value between the target signal and the signal after narrow feature optimization with a range of choices. As one can see from Supplementary Figure 7, for the dipole in the x-direction we observe significant R^2 improvements when going from one to two frequencies and again when going from two to three frequencies as additional narrow features are found. The dipole in the y-direction and the dipole in the z-direction both show constant R^2 values with respect to the number of initial frequencies present. In the case of the y-dipole, the exact result simply has no narrow feature in the observed region. Hence, no change if more frequencies are added. For the dipole in the z-direction, there is only one single narrow feature in the exact result, which is found in BYND with only one input frequency. Therefore, there is also no change in the R^2 for the z-dipole as you increase the number of initial frequencies.

We conclude that the R^2 value is a useful tool for estimating the error associated with the number of SMA input frequencies. We show that, by calculating the R^2 value, one can spot situations where too few frequencies have been selected or where the default $\Delta\omega_{\text{init}}$ is too small to find frequencies which are far away from the SMA input. Beside these two example cases the R^2 value can also be used as a general error estimator without the need of performing a long-time simulation to obtain the exact reference spectrum. One simply calculates the R^2 between target signal and the one obtained after narrow feature optimization. As a rule of thumb, R^2 values should be as large as possible. However, the cyclic nature of our data causes some limitations in using the R^2 value. Strictly speaking, one can not compare R^2 values between time series of different lengths. Some parts of the signal might be reproduced better than other parts, thus, calculating R^2 for one signal length might yield a different value compared to another signal which was cut differently. We also would like to emphasize that, in the case of BYND, only the R^2 calculated between the narrow features of the reference signal and the target signal are meaningful. The full signal constructed from narrow features and a continuum will always give a R^2 close to 1, as fitting the continuum amplitudes will counterbalance errors made in the narrow feature optimization. The fitting of continuum amplitudes is an under-determined system which will always reproduce the correct signal. Only by preconditioning, using for example SMA input frequencies, is one able to extract physically meaningful spectra. Nevertheless, the R^2 value allows us to estimate the quality of the narrow feature optimization without the need of calculating long-time references.

Supplementary Table 1: Selected SMA excitation energies for $Cd_{38}Se_{38}$-ZnPc-DPA-32(NH_2CH_3). The given letters correspond to the letters listed in Supplementary Figure 6.

Dipole signals	excitation energies [eV]				
a)	6.599				
b)	6.599	6.629			
c)	5.524	6.599	6.629		
d)	5.524	5.576	6.599	6.629	
e)	5.524	5.576	6.082	6.599	6.629

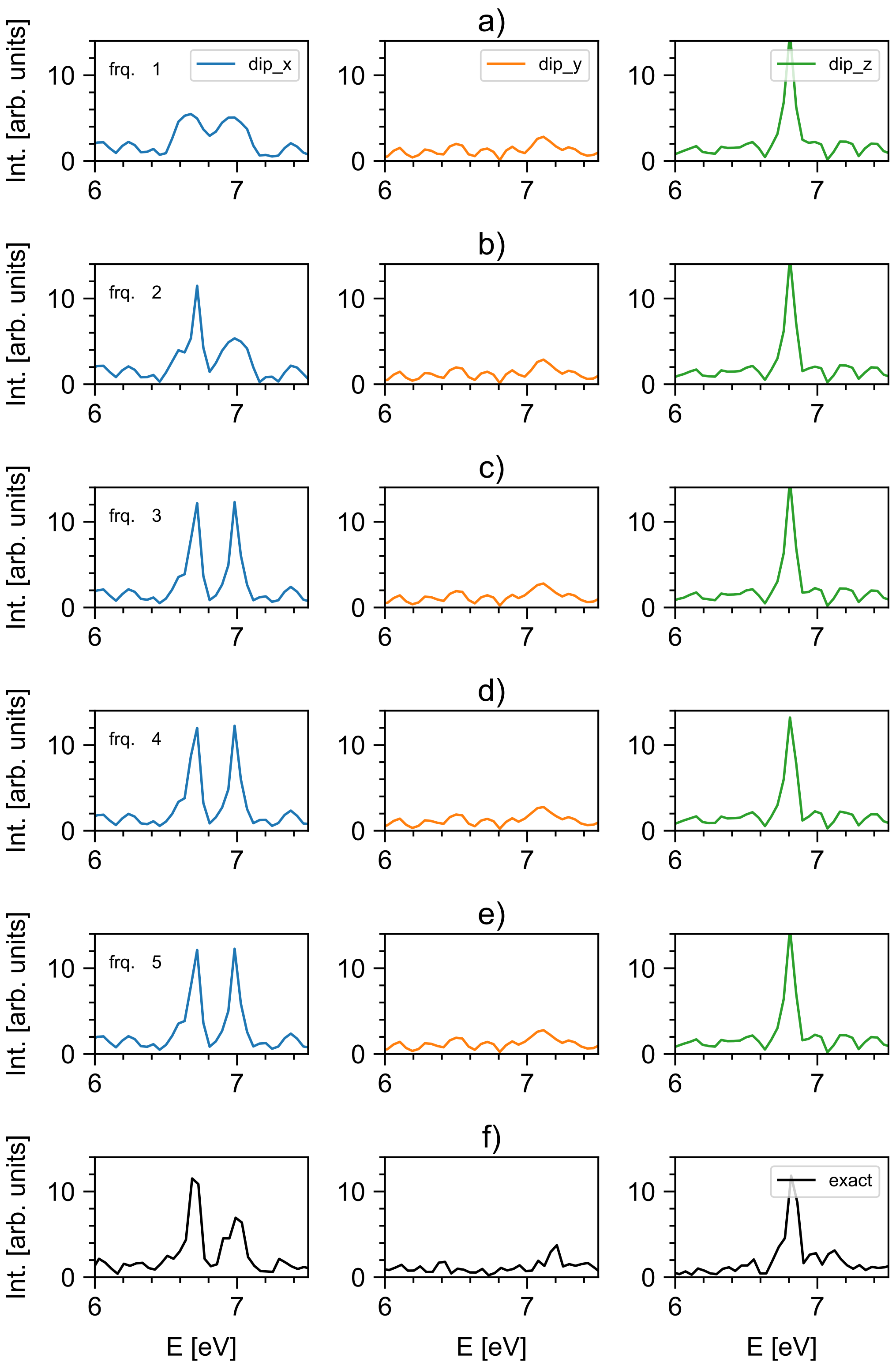

Supplementary Figure 6: Dipole signals of $Cd_{38}Se_{38}$-ZnPc-DPA-32(NH_2CH_3) obtained from BYND using different numbers of input frequencies. a) One initial frequency between 5.5 and 6.7 eV, b) two initial frequencies, c) three frequencies, d) four frequencies and e) five initial frequencies. f) exact reference from a 20,000 time step simulation. We only show the dipole spectrum where the initial electric field vector and the observed dipole are parallel.

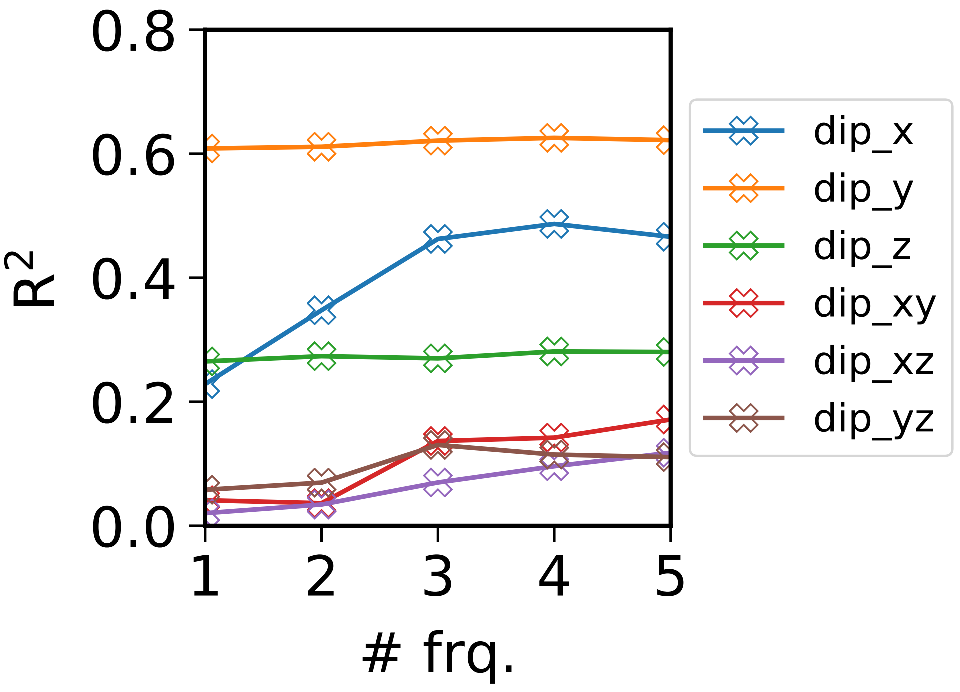

Supplementary Figure 7: Calculated R^2 values between BYND narrow feature energies and the target signal in $Cd_{38}Se_{38}$-ZnPc-DPA-32(NH_2CH_3). Here, we also show results for the dipole signals not parallel to the initial electric field pulse vector (xy, xz and yz).

3 Comparison to Compressed Sensing

Let us assume we want to resolve a certain frequency dependent quantity $g(\omega)$. In general, this quantity can be obtained from the Fourier transform,

$$g(\omega) = \int_{-\infty}^{\infty} dt \sin(\omega t) h(t) \quad , \tag{1}$$

of a corresponding time-dependent function $h(t)$. In Compressed Sensing (CS), one tries to solve the following matrix equation,[2]

$$\mathbf{F}\mathbf{g} = \mathbf{h} \quad . \tag{2}$$

where $\mathbf{g}$ and $\mathbf{h}$ are now the discretized quantities from equation 1 and $\mathbf{F}$ is the $N_\omega \times N_t$ Fourier matrix. The entries of $\mathbf{F}$ can be written as,

$$F_{ij} = \frac{2}{\pi} \Delta\omega \sin(\omega_i t_j) \quad . \tag{3}$$

We are interested in the case where $N_\omega > N_t$, where one is trying to solve equation 3 for the smallest amount of discrete time points possible. This means that our system will be under-determined. Within CS, one addresses this by solving for the sparsest solution for $\mathbf{g}$.[2] This translates to following minimization problem:

$$\min_g \|\mathbf{g}\|_1 \quad \text{subject to} \quad \|\mathbf{F}\mathbf{g} - \mathbf{h}\|_2^2 < \eta \quad . \tag{4}$$

Enforcing the 1-norm in $\mathbf{g}$ thus guarantees that the solution with the largest number of zero elements is selected. Solving equation 4 is also known as basis pursuit denoising (BPDN), where the parameter η accounts for noise in the target signal.[2] The BPDN problem can be cast into following variant,

$$\min_g \frac{1}{2} \|\mathbf{F}\mathbf{g} - \mathbf{h}\|_2^2 + \lambda \|\mathbf{g}\|_1 \quad . \tag{5}$$

which is known as the Lasso problem. Equation 4 and equation 5 are equivalent in that solving one will determine the parameter necessary for the other equation to provide the same solution.[3, 4] In other words, for a given noise parameter η there is a scalar weight λ which yields the same solution.

In the following we will utilize the Lasso formalism of equation 5 to compare our approach with CS. The λ parameter corresponds to the sparsity of our signal, with larger λ values yielding more sparse final spectra. We will apply three different values for λ (= 0.05, 0.01 and 0.001), which have been chosen to yield the best results for our test system $Cd_{38}Se_{38}$-ZnPc-32(NH_2CH_3). Just as we do with our algorithm, we will apply CS to short-time dynamics TDDFT signals with length varying from 500 to 5,000 time steps. We assess the performance of each method by comparing how accurately they reproduce the dipole spectrum in the x-, y- and z-direction. Here, a dipole in the x-direction means that we apply an electric field pulse along that direction and also measure the dipole oscillation in the same direction. Our results are shown in Supplementary Figures 8-12.

For 500, 1,000, and 1,500 time steps (Supplementary Figures 8, 9, and 10), CS is able to correctly identify the main narrow spectral features with all three values of λ. However, CS also predicts multiple additional narrow features in the region where the exact spectrum has the continuum. This clearly indicates the downside of CS selecting only sparse solutions. On the other hand, our approach yields both the narrow features and the continuum with high accuracy for these short signals.

The shortcomings of CS are still present when one includes more time steps. At 3,000 time steps (Supplementary Figure 11), the best results for CS are obtained with a λ value of 0.001. For $\lambda = 0.005$ CS predicts almost no features in the continuum region, and completely omits the y-dipole component at 3.5 eV. A λ value of 0.001 gives the best CS result, though it still incorrectly places high-amplitude features in the continuum region.

At 5,000 time steps, for $\lambda = 0.001$ the additional "false" narrow features are reduced in their intensity. $\lambda = 0.0001$ now starts to accurately reproduce the dipole spectrum. However, $\lambda = 0.0001$ is so small that we argue it amounts to only very weakly enforcing the sparsity constraint. With 5,000 time steps, one now has sufficient data that an acceptable solutions is achieved and even here, we observe that CS yields a continuum region that is interrupted by gaps.

In comparison, our approach reproduces the dipole spectrum with very high accuracy for all signal lengths. With only 1,500 time steps (Supplementary Figure 10) our approach yields almost perfect agreement with the long time simulation.

We conclude that CS is able to correctly reproduce the narrow features in our test system, but the quasi-continuum will lead to the emerging of additional "false" narrow features which are still present even if one increases the target signal length to 5,000 time steps. In addition, CS is highly dependent on the sparsity parameter, and even with an ideal parameter choice CS only correctly places the target narrow features when one employs signal lengths of at least 3,000 time steps. Meanwhile, our approach is able to reproduce the dipole spectrum with only 500 time steps of simulation time.

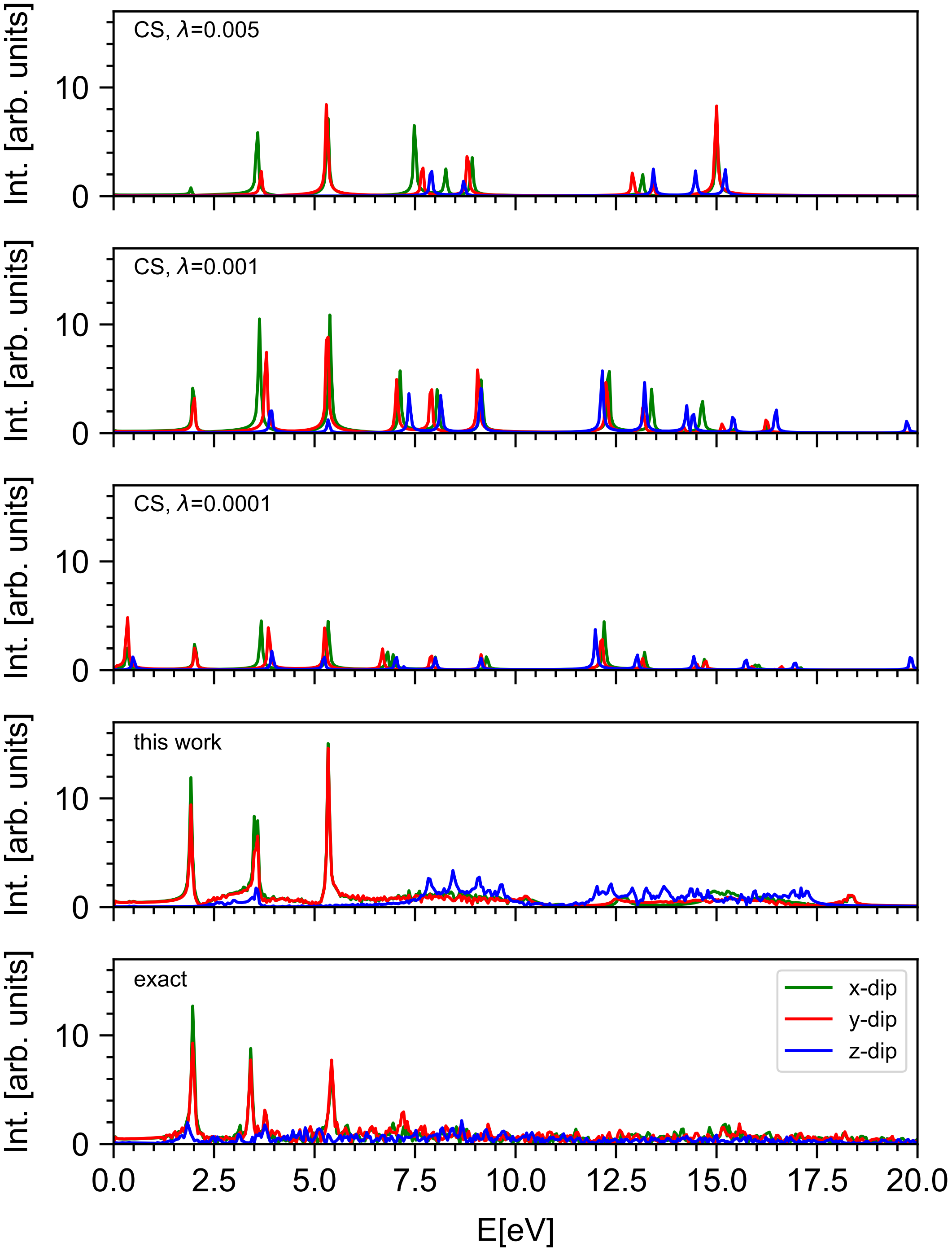

Supplementary Figure 8: Comparison of Compressed Sensing (CS) with our approach (BYND) and the Fourier transform of a long-time signal (exact) with 20,000 time steps. We only show the dipole spectrum for the x-, y- and z-direction. By this, we mean that we apply an electric field pulse in a certain direction and measure the dipole oscillation along the direction of the applied field. Compressed Sensing results and the results of our approach have been obtained for a simulation time of 500 time steps.

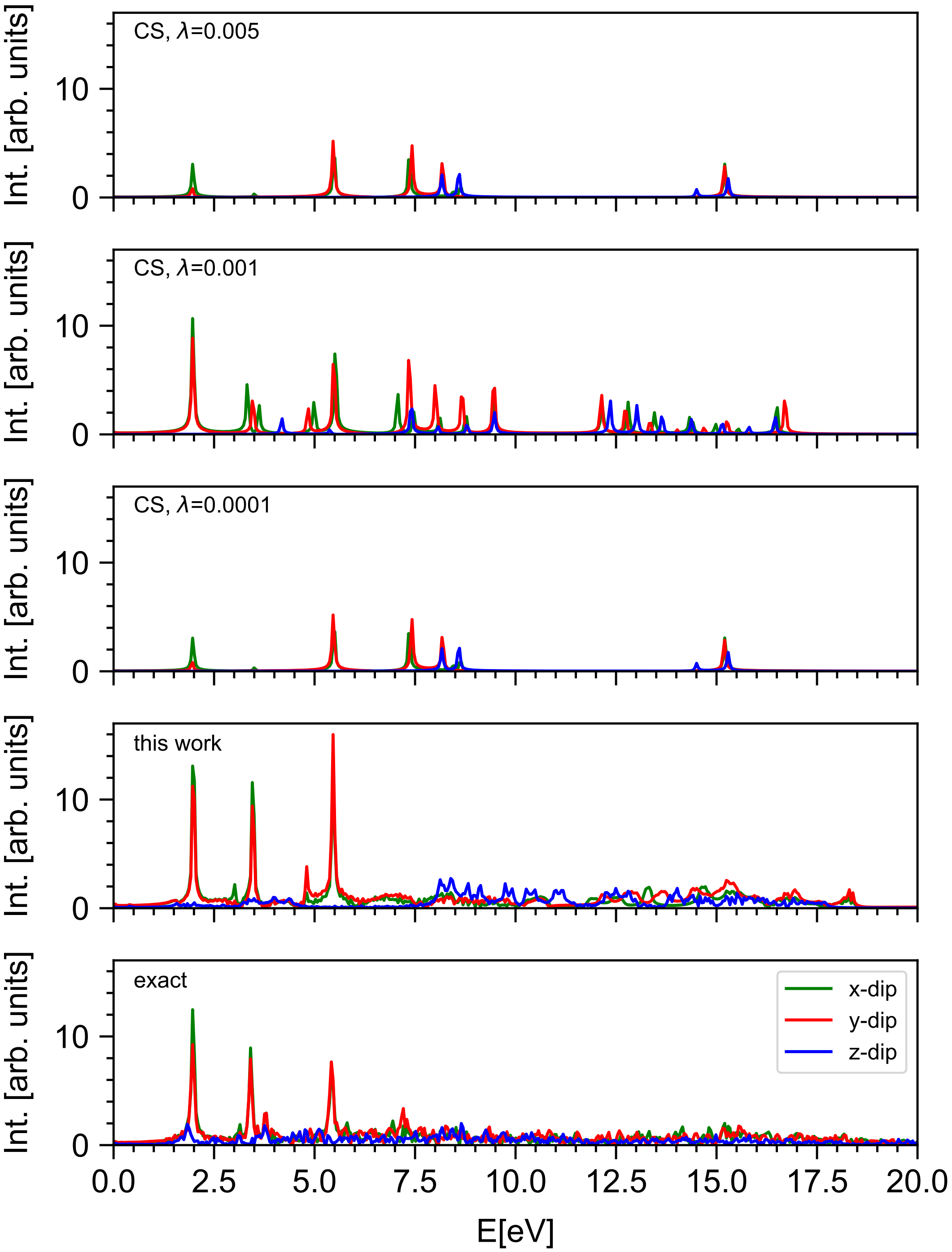

Supplementary Figure 9: Comparison of Compressed Sensing (CS) with our approach (BYND) and the Fourier transform of a long-time signal (exact) with 20,000 time steps. We only show the dipole spectrum for the x-, y- and z-direction. By this, we mean that we apply an electric field pulse in a certain direction and measure the dipole oscillation along the direction of the applied field. Compressed Sensing results and the results of our approach have been obtained for a simulation time of 1,000 time steps.

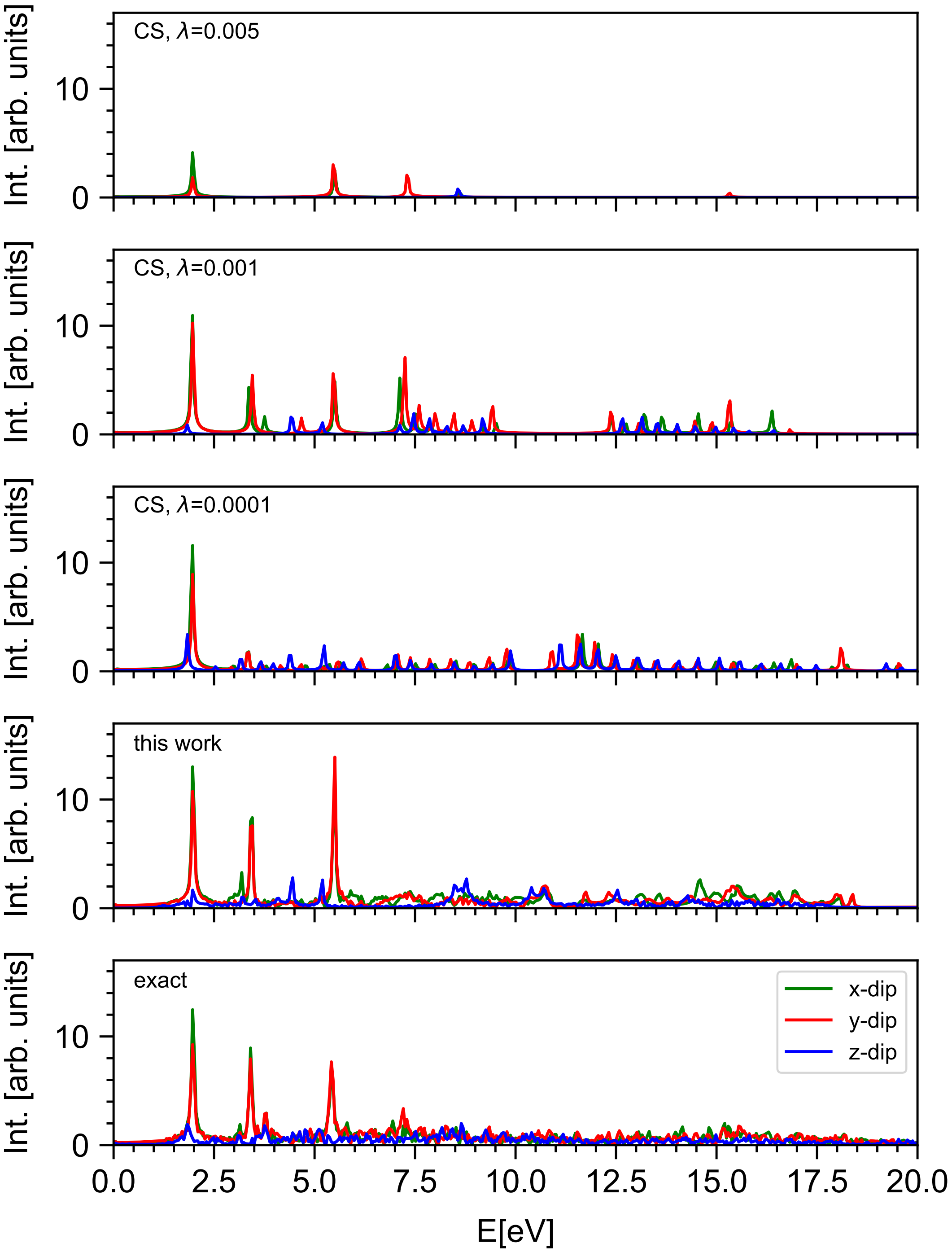

Supplementary Figure 10: Comparison of Compressed Sensing (CS) with our approach (BYND) and the Fourier transform of a long-time signal (exact) with 20,000 time steps. We only show the dipole spectrum for the x-, y- and z-direction. By this, we mean that we apply an electric field pulse in a certain direction and measure the dipole oscillation along the direction of the applied field. Compressed Sensing results and the results of our approach have been obtained for a simulation time of 1,500 time steps.

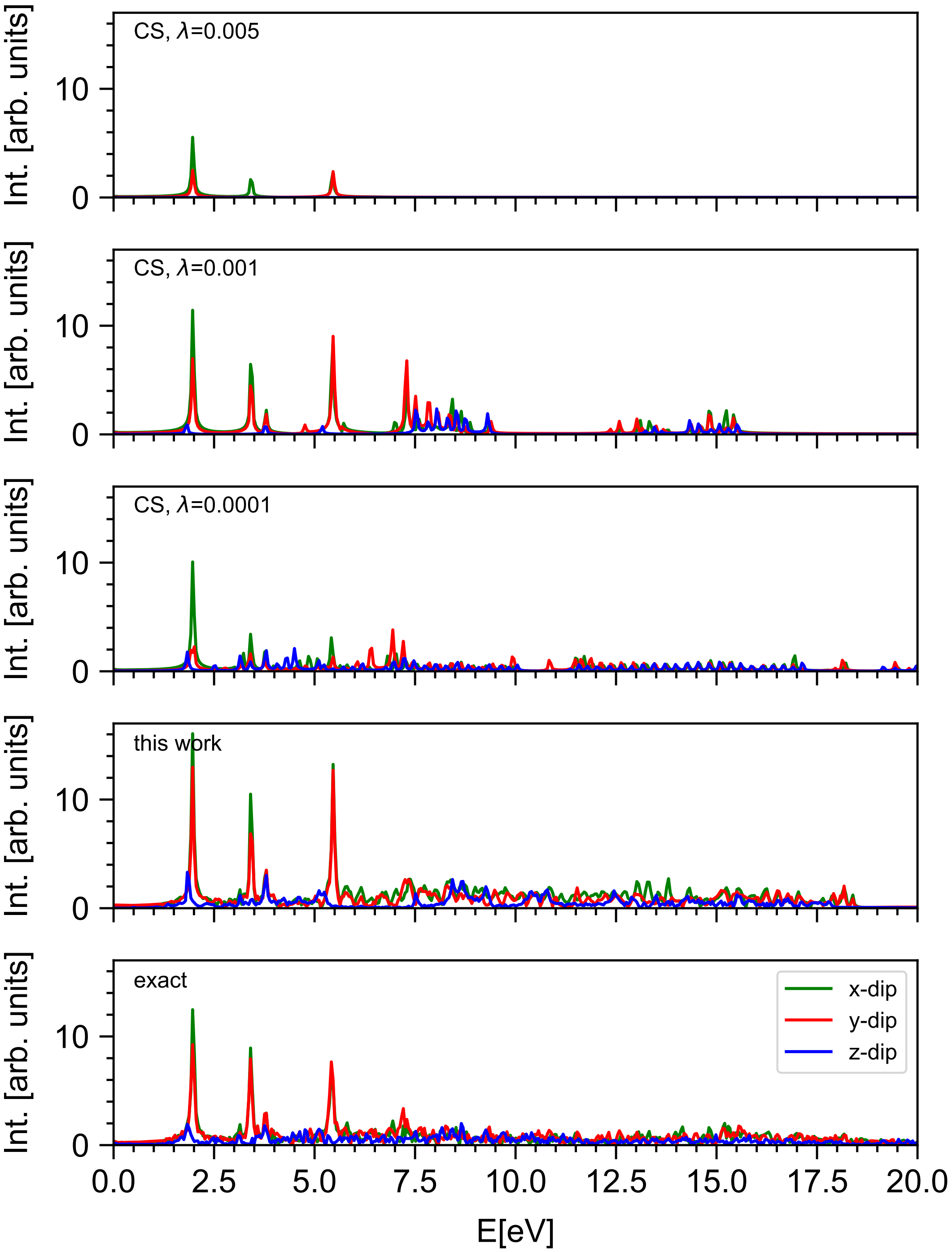

Supplementary Figure 11: Comparison of Compressed Sensing (CS) with our approach (BYND) and the Fourier transform of a long-time signal (exact) with 20,000 time steps. We only show the dipole spectrum for the x-, y- and z-direction. By this, we mean that we apply an electric field pulse in a certain direction and measure the dipole oscillation along the direction of the applied field. Compressed Sensing results and the results of our approach have been obtained for a simulation time of 3,000 time steps.

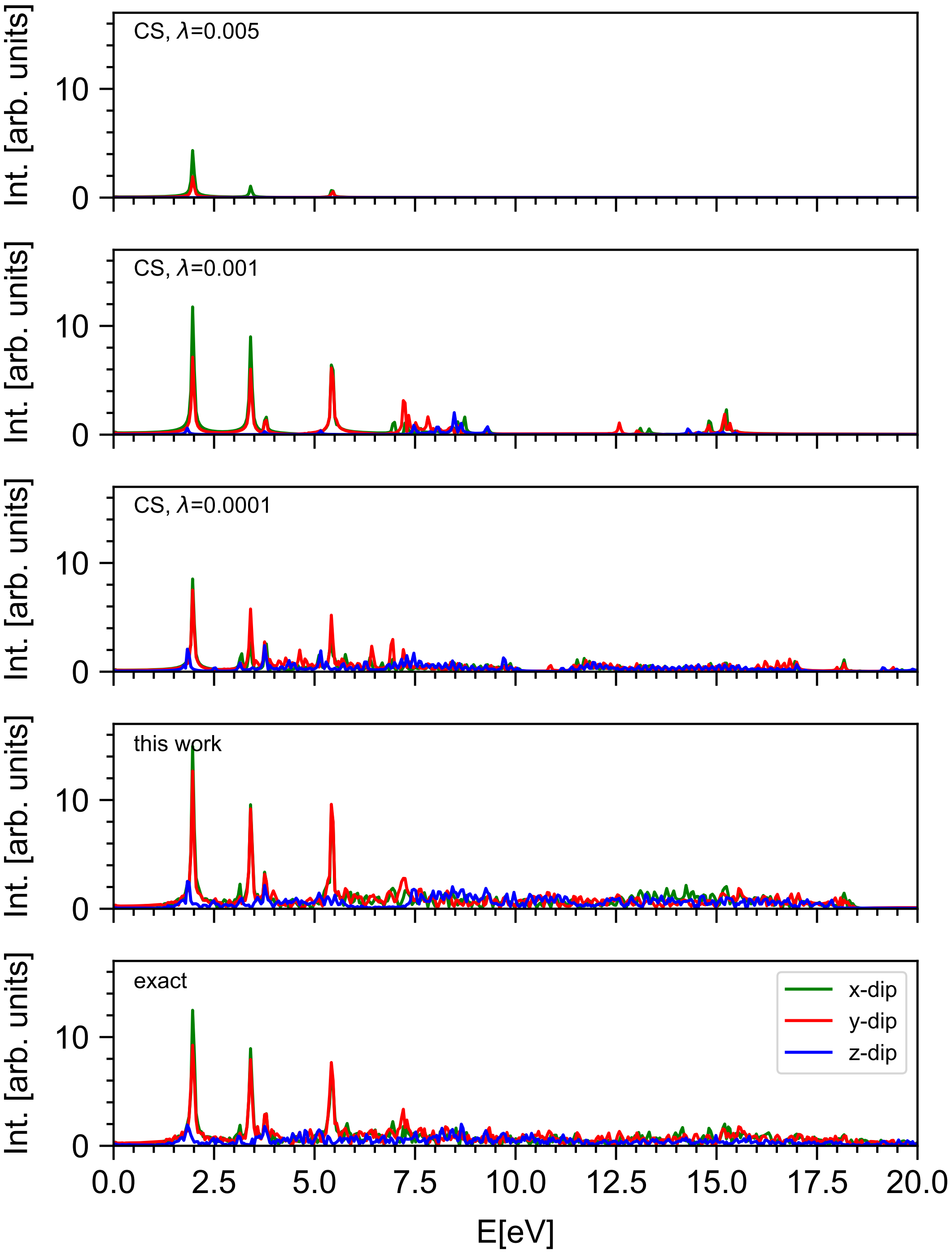

Supplementary Figure 12: Comparison of Compressed Sensing (CS) with our approach (BYND) and the Fourier transform of a long-time signal (exact) with 20,000 time steps. We only show the dipole spectrum for the x-, y- and z-direction. By this, we mean that we apply an electric field pulse in a certain direction and measure the dipole oscillation along the direction of the applied field. Compressed Sensing results and the results of our approach have been obtained for a simulation time of 5,000 time steps.

4 Influence of ridge regression parameter

An important step of our algorithm is the determination of the correct amplitudes for each narrow feature in the spectrum. For this purpose we make use of ridge regression,[5] also known as Tikhonov regularization:[6]

$$\min \frac{1}{n} \sum_i ||y_i^{\lambda\mu} - f_{\lambda\mu}^{\text{sparse}}(A_k^{\lambda\mu}, \omega_k, t_i)||_2^2 + \alpha_{\text{sparse}} ||f^{\text{sparse}}||_2^2 \quad . \tag{6}$$

The regularization parameter α_{sparse} enforces solutions with smaller norms and thus a different choice of α_{sparse} can in principle affect the final result. In the following, we show how robust our algorithm is regarding the choice of α_{sparse}. For this purpose we use our $Cd_{38}Se_{38}$-ZnPc-32(NH_2CH_3) test system and vary α_{sparse} between 10 and 100. We use a target signal length of 1,000 time steps and use 11 frequencies for the initial guess, analogous to Supplementary Figure 2c. Supplementary Figure 13 summarizes our findings.

For $\alpha_{\text{sparse}} = 10$, 20 and 30 our method accurately reproduces all three narrow features. Moreover, the anisotropy between the different dipole directions is roughly correct for each narrow feature in these spectra. For α_{sparse} values of 50 and 100, however, we observe that the third narrow feature is split into two bright closely-spaced excitations. This arises from the penalization term in ridge regression, which penalizes solutions with a few large amplitudes over those with several smaller values. Therefore, we suggest using a default value of $\alpha_{\text{sparse}} = 20$ for this part of our method and recommend to not use values larger than 30 as otherwise unwanted spectral features could occur.

In order to obtain the amplitudes for the continuum, we perform a second ridge regression step after optimization of the narrow features,

$$\min \frac{1}{n} \sum_i ||y_i^{\text{cont},\lambda\mu} - f_{\lambda\mu}^{\text{cont}}(A_k^{\lambda\mu}, \omega_k, t_i)||_2^2 + \alpha_{\text{cont}} ||f^{\text{cont}}||_2^2 \quad . \tag{7}$$

Again, we explore the choice of the corresponding ridge regression parameter α_{cont} by varying from 10 to 100. Supplementary Figure 14 shows our results. As one can see, the continuum region is qualitatively reproduced with any choice of α_{cont}. However, when working on the amplitudes of the continuum region we can safely expect that all frequencies contribute roughly equally. Therefore, it is desirable to use high α_{cont} values in order to properly penalize solutions with large amplitudes. Hence, to obtain the correct amplitudes for the continuum region we suggest performing this second ridge regression with an α value of at least 50 or above. As a default value we choose 100 for our calculations.

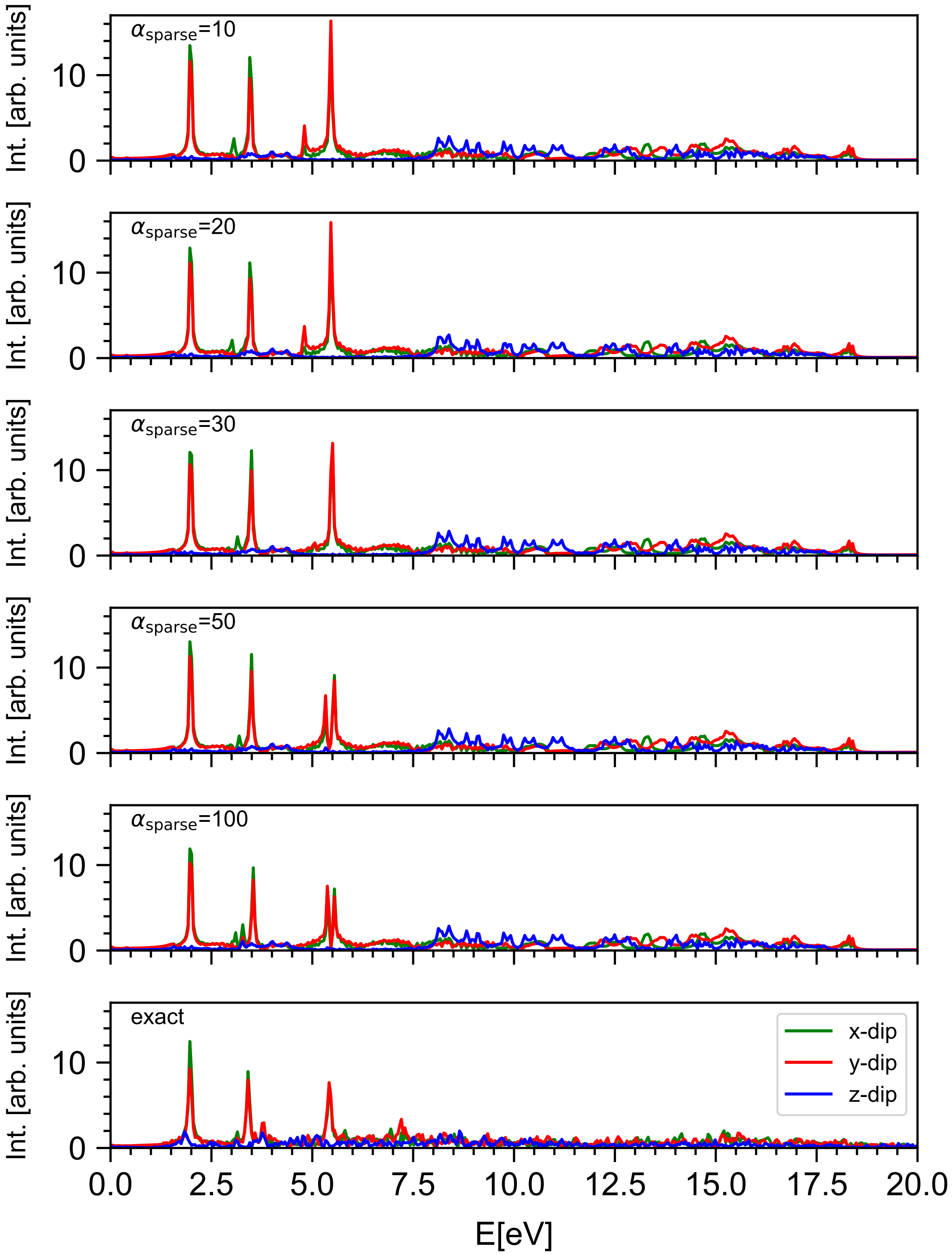

Supplementary Figure 13: We show the influence of the α_{sparse} parameter in our ridge regression step. We observe that high α_{sparse} values lead to a splitting of the third narrow feature. For these calculations, we employ 1,000 time steps and an initial guess threshold that yields 11 initial guess frequencies.

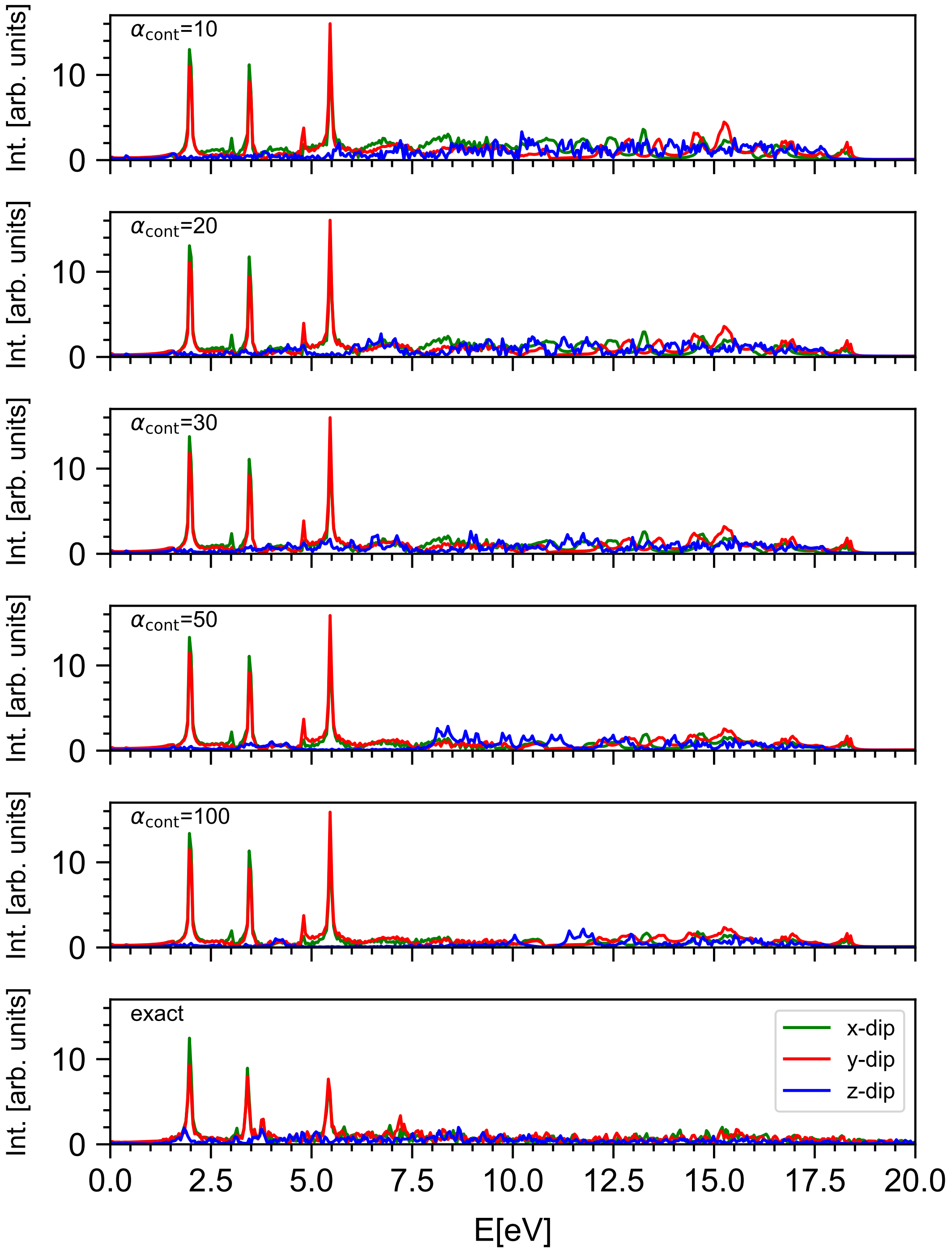

Supplementary Figure 14: We show the influence of the α_{cont} parameter in our ridge regression step for the continuum amplitudes. We observe that all α_{cont} values qualitatively reproduce the continuum region. For these calculations, we employ 1,000 time steps and an initial guess threshold that yields 11 initial guess frequencies.

5 Small and medium sized molecules

To demonstrate the capability of our approach for other types of systems, we perform BYND calculations on benzene, p3b2 and f-cororene (Supplementary Figure 15). Benzene is a small organic molecule which shows a spectrum consisting of distinct, well separated excitations. Thus, benzene serves as a test system for sparse signals. P3b2 and f-cororene are organic dye molecules consisting of 136 and 160 atoms, respectively. Due to their size, we expect to see significant continuum excitations, and in fact we see 500 excitations for p3b2 and 715 excitations for f-cororene within 5 eV according to our SMA simulation. Thus, p3b2 and f-cororene are excellent molecular target systems for our approach.

From Supplementary Figure 15, we see that with only 500 time steps the excitation spectrum of benzene is reproduced with good accuracy. Including more data points improves the intensities further, up to 1,500 time steps where we obtain almost perfect accuracy. The spectrum of p3b2 is well reproduced at 500 time steps, with the exception that the narrow features are slightly shifted to higher excitation energies. The spectral features at around 3.5 eV have also incorrectly collapsed to a single peak. This latter issue persists up to 5,000 time steps. Additionally, at 1,500 time steps we observe the appearance of a shoulder peak around 3 eV. This arises from the algorithm approximating several closely spaced peaks as one single bright feature. F-cororene is a challenging system as it has several bright features within the first 4 eV that are superimposed over a large quasi-continuum. However, with only 1,500 time steps we start to observe good accuracy with respect to the exact result. At 5,000 time steps we are able to achieve near-perfect agreement.

All TDDFT calculations have been carried out using the FHIaims[7] program. Exchange-correlation interactions have been treated using the PBE[8] functional. *Tight tier1* settings have been used for the integration grid and basis set. All RT-TDDFT[9] calculations have been performed with a time step of 0.2 a.u. and an electric field strength of 0.01 a.u.. Total simulation time was 4,000 a.u.. For benzene, we instead used a time step of 0.1 au and a total simulation time of 2,500 a.u..

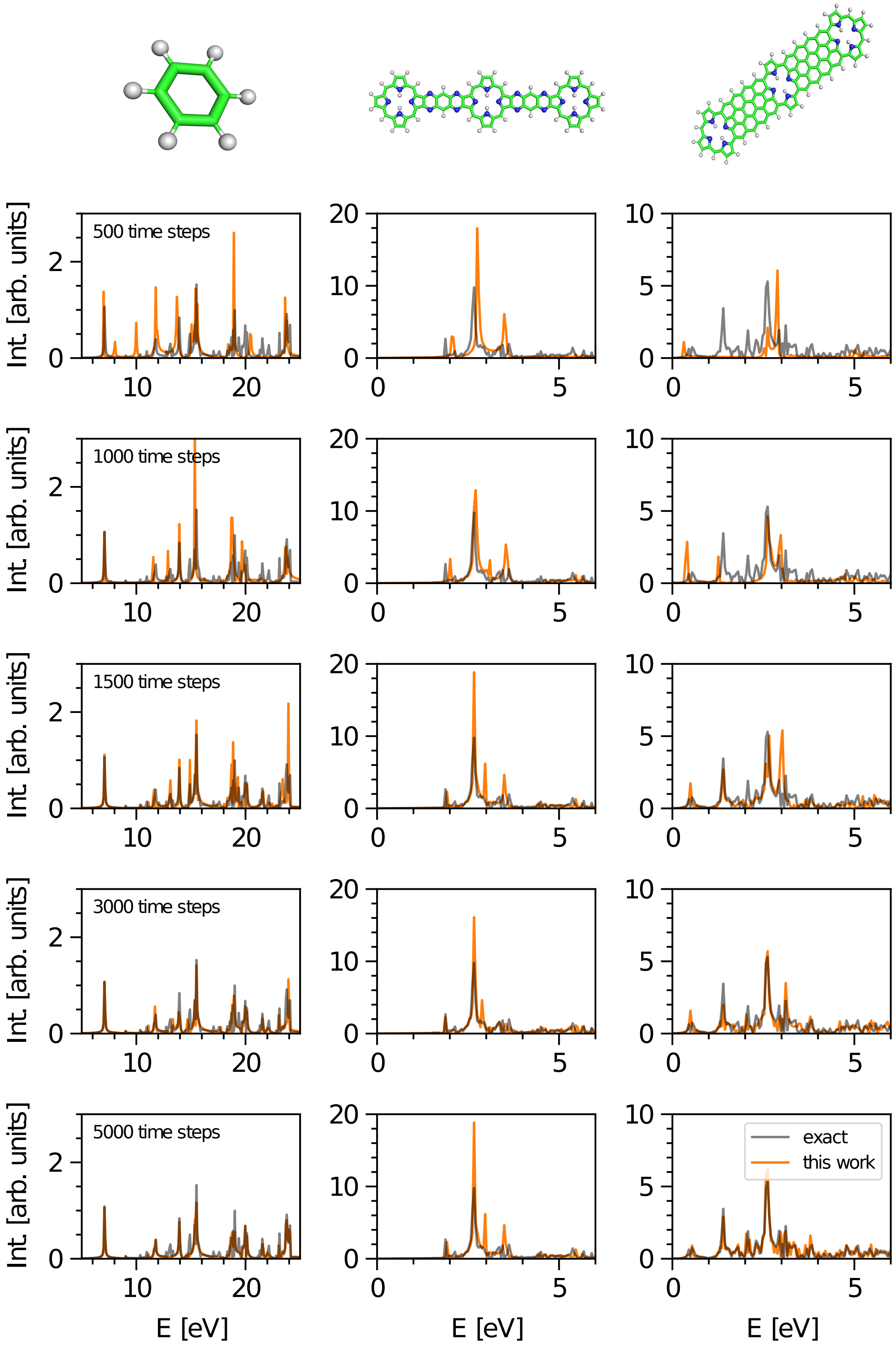

Supplementary Figure 15: Excitation spectrum of benzene (left), p3b2 (middle) and f-cororene (right).

6 Initial guess frequencies

$Cd_{38}Se_{38}$-ZnPc-32(NH_2CH_3)

The initial search radius was set to $\Delta\omega_{\text{init}} = 0.05$ a.u.. All following search grid settings used a value of $\Delta\omega_{\text{init}} = 0.001$ a.u.. Supplementary Table 2 lists the initial guess frequencies which we obtained from the SMA calculation.

$Cd_{38}Se_{38}$-ZnPc-DPA-32(NH_2CH_3)

The initial search radius was set to $\Delta\omega_{\text{init}} = 0.065$ a.u. for the 500 time step simulation. All other simulations used an initial search grid radius of $\Delta\omega_{\text{init}} = 0.05$ a.u.. All following line-search steps use $\Delta\omega = 0.001$ a.u.. Supplementary Table 3 lists the initial guess frequencies which we used for the 500, 1,000 and 1,500 time step spectrum. Supplementary Table 4 lists the initial guess frequencies which we used for the 3,000 and 5,000 time step spectrum. Only by using more frequencies are we able to resolve the spectral features at around 7 eV, as otherwise there are not enough frequencies available in this area.

$Cd_{33}Se_{33}/Zn_{93}S_{93}$-2(ZnPc)

The initial search radius was set to $\Delta\omega_{\text{init}} = 0.05$ a.u.. All following search grid settings used a value of $\Delta\omega_{\text{init}} = 0.001$ a.u.. Supplementary Table 5 lists the initial guess frequencies which we obtained from the SMA calculation.

$Cd_{33}Se_{33}/Zn_{93}S_{93}$-2(ZnPc)-DPA

The initial search radius was set to $\Delta\omega_{\text{init}} = 0.05$ a.u.. All following search grid settings used a value of $\Delta\omega_{\text{init}} = 0.001$ a.u.. Supplementary Table 7 lists the initial guess frequencies which we obtained from the SMA calculation.

ZnPc j-aggregate

The initial search radius was set to $\Delta\omega_{\text{init}} = 0.05$ a.u.. All following search grid settings used a value of $\Delta\omega_{\text{init}} = 0.001$ a.u.. Supplementary Table 11 lists the initial guess frequencies which we obtained from the SMA calculation.

cis-Ru@anatase(101)

The initial search radius was set to $\Delta\omega_{\text{init}} = 0.05$ a.u.. All following search grid settings used a value of $\Delta\omega_{\text{init}} = 0.001$ a.u.. Supplementary Table 12 lists the initial guess frequencies which we obtained from the SMA calculation.

ZnPc@Si(111)

The initial search radius was set to $\Delta\omega_{\text{init}} = 0.05$ a.u.. All following search grid settings used a value of $\Delta\omega_{\text{init}} = 0.001$ a.u.. Supplementary Table 13 lists the initial guess frequencies which we obtained from the SMA calculation.

Zinc porphyirne @ carbon nanotube

The initial search radius was set to $\Delta\omega_{\mathrm{init}}$ = 0.05 a.u.. All following search grid settings used a value of $\Delta\omega_{\mathrm{init}}$ = 0.001 a.u.. Supplementary Table 14 lists the initial guess frequencies which we obtained from the SMA calculation.

Benzene

The initial search radius was set to $\Delta\omega_{\mathrm{init}}$ = 0.05 a.u.. All following search grid settings used a value of $\Delta\omega_{\mathrm{init}}$ = 0.05 a.u.. Supplementary Table 8 lists the initial guess frequencies which we obtained from the SMA calculation.

P3b2

The initial search radius was set to $\Delta\omega_{\mathrm{init}}$ = 0.05 a.u.. All following search grid settings used a value of $\Delta\omega_{\mathrm{init}}$ = 0.05 a.u.. Supplementary Table 9 lists the initial guess frequencies which we obtained from the SMA calculation.

F-cororene

The initial search radius was set to $\Delta\omega_{\mathrm{init}}$ = 0.05 a.u.. All following search grid settings used a value of $\Delta\omega_{\mathrm{init}}$ = 0.05 a.u.. Supplementary Table 10 lists the initial guess frequencies which we obtained from the SMA calculation.

Supplementary Table 2: $Cd_{38}Se_{38}$-ZnPc-32(NH_2CH_3): Initial excitation energies.

init. exc. energies [eV]	1.622	1.845	1.899	1.905	1.951	2.019	2.400	2.405	3.861	3.889	4.830
initial search radius [a.u.]	0.050										
search radius [a.u.]	0.001										

Supplementary Table 3: $Cd_{38}Se_{38}$-ZnPc-DPA-32(NH_2CH_3): Initial excitation energies for 500, 1,000 and 1,500 time step spectra.

init. exc. energies [eV]	2.060	2.063	2.117	2.120	4.242	4.683	6.599	6.629
initial search radius [a.u.]	0.065/0.050							
search radius [a.u.]	0.001							

Supplementary Table 4: $Cd_{38}Se_{38}$-ZnPc-DPA-32(NH_2CH_3): Initial excitation energies for 3,000 and 5,000 time step signals.

init. exc. energies [eV]	2.060	2.063	2.117	2.120	3.687	3.742	3.774	4.242	4.253
	4.683	5.524	6.599	6.629	8.079				
initial search radius [a.u.]	0.050								
search radius [a.u.]	0.001								

Supplementary Table 5: $Cd_{33}Se_{33}/Zn_{93}S_{93}$-2(ZnPc): Initial excitation energies.

init. exc. energies [eV]	1.527	1.712	1.739	2.038	2.049	2.065	2.071	2.133	2.142
	2.316	2.359	2.395	2.604	3.023	3.110	4.923		
initial search radius [a.u.]	0.050								
search radius [a.u.]	0.001								

Supplementary Table 6: $Cd_{33}Se_{33}/Zn_{93}S_{93}$-2(ZnPc)-DPA: Initial excitation energies for 500 time steps.

init. exc. energies [eV]	2.060	2.063	2.117	2.120	3.687	3.742	3.774	4.242	4.683
	6.599	6.629							
initial search radius [a.u.]	0.050								
search radius [a.u.]	0.001								

Supplementary Table 7: $Cd_{33}Se_{33}/Zn_{93}S_{93}$-2(ZnPc)-DPA: Initial excitation energies for 1,500 time steps and more.

init. exc. energies [eV]	2.060	2.063	2.117	2.120	3.687	3.742	3.774	4.242	4.253
	4.683	5.524	6.599	6.629	8.079				
initial search radius [a.u.]	0.050								
search radius [a.u.]	0.001								

Supplementary Table 8: Benzene: Initial excitation energies.

init. exc. energies [eV]	7.053	7.053	7.720	7.720	11.916	11.916	13.331	13.331	15.189
	15.189	15.369	15.369	17.979	17.979	18.360	18.724	18.724	23.407
	23.407	30.245	33.783	34.403	34.403	34.743	34.743	35.326	35.326
initial search radius [a.u.]	0.050								
search radius [a.u.]	0.001								

Supplementary Table 9: P3b2: Initial excitation energies.

init. exc. energies [eV]	2.139	2.672	3.127	3.238
initial search radius [a.u.]	0.050			
search radius [a.u.]	0.001			

Supplementary Table 10: F-cororene: Initial excitation energies.

init. exc. energies [eV]	0.634	1.608	2.852	2.966	3.244	12.604
initial search radius [a.u.]	0.050					
search radius [a.u.]	0.001					

Supplementary Table 11: ZnPc j-aggregate: Initial excitation energies.

init. exc. energies [eV]	1.592	1.597	1.622	1.845	1.872	1.899
	1.905	1.951	2.019	2.400	2.405	2.610
	3.861	3.889	4.830	4.939		
initial search radius [a.u.]	0.050					
search radius [a.u.]	0.001					

Supplementary Table 12: cis-Ru@anatase(101): Initial excitation energies.

init. exc. energies [eV]	0.914	0.922	0.980	1.426	2.474	2.528
	2.558	3.766	3.791	3.859		
initial search radius [a.u.]	0.050					
search radius [a.u.]	0.001					

Supplementary Table 13: ZnPc@Si(111): Initial excitation energies.

init. exc. energies [eV]	1.611	1.758	1.769	1.777	1.791	1.796
	1.801	1.839	1.875	1.875	1.888	1.908
	1.916	1.924	1.932	1.935	1.973	2.049
	2.057	3.132				
initial search radius [a.u.]	0.050					
search radius [a.u.]	0.001					

Supplementary Table 14: Zinc porphyirne @ carbon nanotube: Initial excitation energies.

init. exc. energies [eV]	0.759	0.803	1.867	1.872	1.992	2.011
	2.204	2.218	2.816	2.863	2.933	2.961
initial search radius [a.u.]	0.050					
search radius [a.u.]	0.001					

7 Different DFT functionals

Here, we demonstrate the robustness of our approach regarding variation of the RT-TDDFT signal. To reiterate, we use the SMA as an initial guess of an underlying TDDFT calculation using the PBE functional. For the RT-TDDFT calculations, we will test three different functional approximations: VWN[10], PBE[8] and PBE0[11, 12]. The first two functionals are LDA and GGA based, respectively, while the latter belongs to the class of hybrid functionals. This will give three different RT-TDDFT signals, where only the propagation with the PBE functional will match the functional we use for the SMA simulation. From Figure 16-18, we see that in all cases our approach is able to yield accurate results. The most accurate results can be obtained for the PBE case, which is not surprising as both the RT-TDDFT and SMA simulations have been obtained with the same functional. Nevertheless, this comparison clearly shows that the initial guess does not necessarily need to be from the same functional which is used in the RT-TDDFT simulation. One could start with an LDA or GGA approximation and improve the SMA results with a real-time propagation using a hybrid functional.

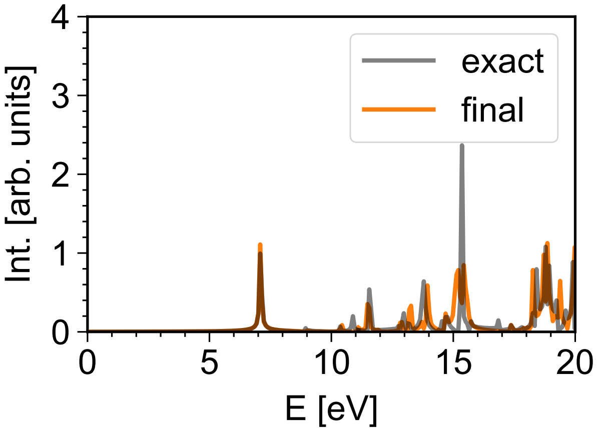

Supplementary Figure 16: Absorption spectrum of Benzene. We use the form of the exchange-correlation potential given by Vosko, Wilk, and Nusair (VWN) for the RT-TDDFT run. The initial guess from the SMA is calculated using the PBE functional. The short time RT-TDDFT propagation has a length of 1,500 time steps.

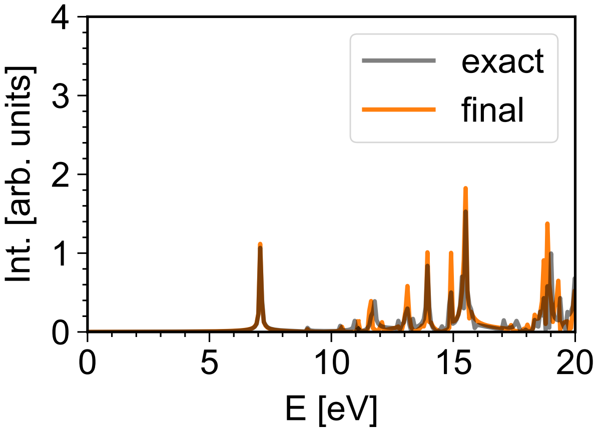

Supplementary Figure 17: Absorption spectrum of Benzene. We use the PBE functional for both the RT-TDDFT run and the SMA initial guess. The short time RT-TDDFT propagation has a length of 1,500 time steps.

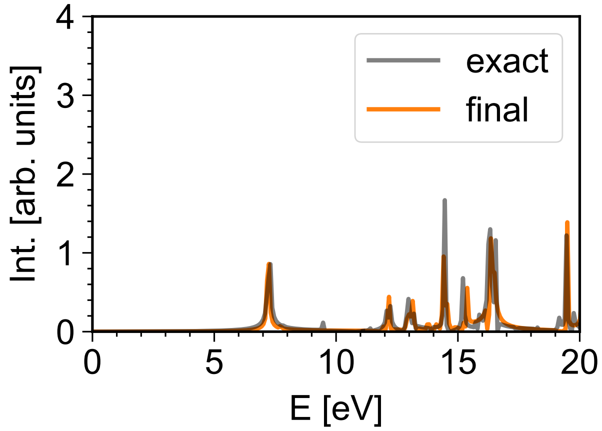

Supplementary Figure 18: Absorption spectrum of Benzene. We use the PBE0 functional for the RT-TDDFT run. The initial guess from the SMA is calculated using the PBE functional. The short time RT-TDDFT propagation has a length of 1,500 time steps.

8 Padé approximation

Here, we compare BYND with the Padé approximation [13]. Generally speaking, the Fourier transform of a time dependent signal can be expressed in form of a power series expansion. The Padé approximation rearranges such an expansion to a ratio of two power series. The remaining task is to find the so called Padé coefficients. One advantage of the Padé approximation is that the transformation into a ratio of two power series usually leads to accelerated convergence of the spectrum. For more details, the interested reader is referred to reference [13] and [14]. Here, we compare the convergence of the final excitation spectrum with respect to the number of time steps for BYND and the Padé approximation (Supplementary Figure 19). We compare both findings with the near-exact solution obtained from the Fourier transform of a 20,000 time step long RT-TDDFT signal. Here, we employ $Cd_{38}Se_{38}$-ZnPc-32(NH_2CH_3) as our test system. As one can see from Supplementary Figure 19, the Padé approximation

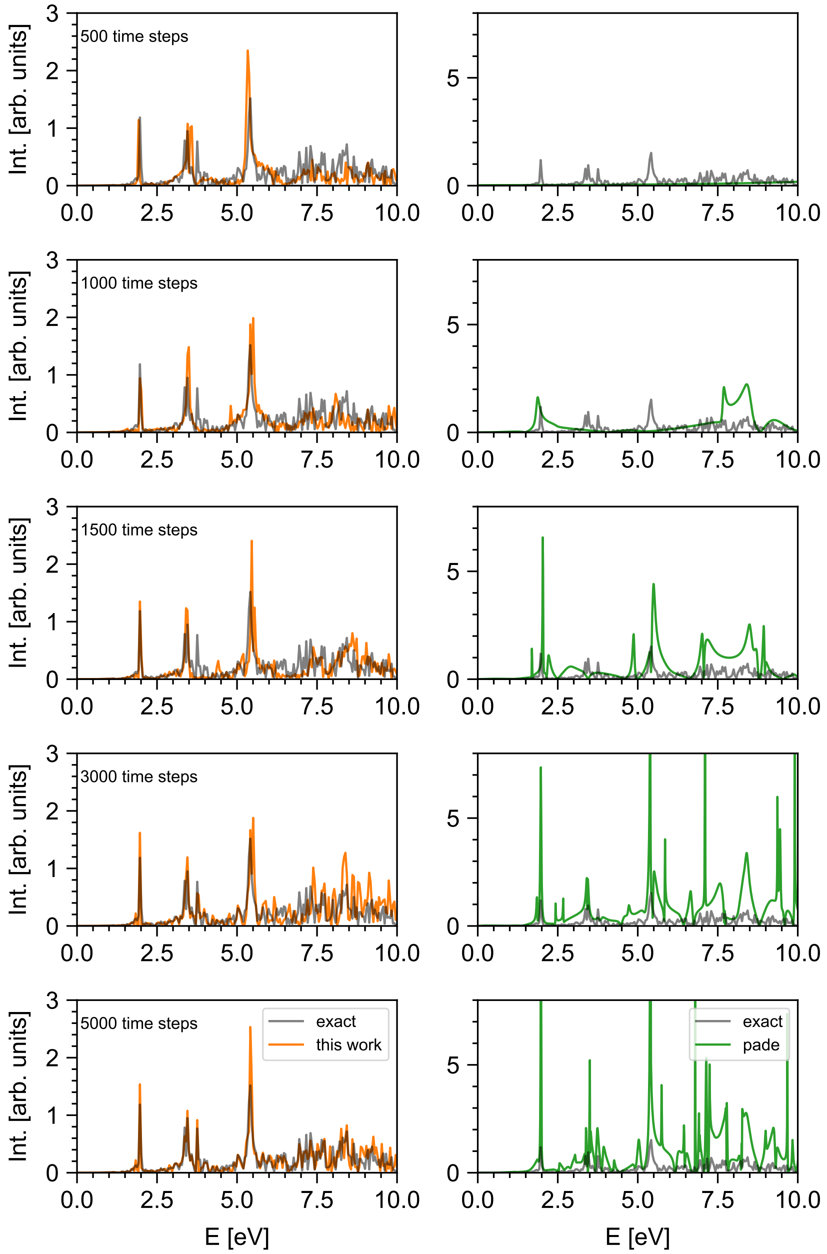

Supplementary Figure 19: Comparison of our approach (left) with the Padé approximation (right). Our "exact" reference is the Fourier transform of a 20,000 time step RT-TDDFT signal.

starts to find narrow features at around 1,500 time steps, as both the feature at roughly 1.9 eV and 5.5 eV are nearly correctly reproduced. At 3,000 time steps, the Padé approximation is additionally able to find the narrow feature at roughly 2.9 eV. For all signal lengths, the Padé approximation has significant difficulty reproducing the correct intensities. Furthermore, the Padé approximation creates bright spectral artifacts in the continuum region. This effect persists even at signal lengths of 5,000 time steps. In contrast, our approach already yields very good results with only 500 time steps.

9 Narrow feature position error estimation

To estimate the error between our BYND results and long-time dynamics we calculate the standard error of the mean of the narrow features, given by:

$$\sigma^{-} = \frac{\sigma}{\sqrt{n}} \quad , \tag{8}$$

where σ is the standard deviation of the difference between the narrow features of BYND and the exact result and n is the number of samples. A specific problem arises when the split between two bright features is very small. Recall that, when one provides BYND with insufficient data points, it may not be able to fully resolve closely spaced narrow features, instead approximating them as only one single peak. Here, BYND shows at least one fewer narrow feature than the reference method. In this case, we compute the error of this narrow feature relative to the closest narrow feature predicted by BYND. For our three nanocrystal systems $Cd_{38}Se_{38}$-ZnPc-32(NH_2CH_3), $Cd_{38}Se_{38}$-ZnPc-DPA-32(NH_2CH_3) and $Cd_{33}Se_{33}/Zn_{93}S_{93}$-2(ZnPc), Supplementary Table 15 lists the calculated standard errors. With only 1,500 time steps, we achieve meV-accuracy. Only for $Cd_{38}Se_{38}$-ZnPc-DPA-32(NH_2CH_3) do we observe an error above 100 meV. This is one of the aforementioned cases, where BYND predicts three narrow features while the exact spectrum contains four narrow features. Here, 1,500 time steps is not enough to fully resolve all the details of the spectrum. Upon using at least 3,000 time steps, this error drops significantly. In fact, 3,000 time steps reduces all errors below 25 meV as BYND convergences towards the "exact" result.

Supplementary Table 15: Standard error of the mean (SEM) for three test crystals. The error compares the results from BYND with the exact results from a RT-TDDFT run. For BYND, we compare results using a time signal of 1,500 and 3,000 time steps. The exact reference was obtained after Fourier transform of a 20,000 time step long RT-TDDFT dipole signal.

System	SEM 1.5k [eV]	SEM 3.0k [eV]
$Cd_{38}Se_{38}$-ZnPc-32(NH_2CH_3)	0.089	0.022
$Cd_{38}Se_{38}$-ZnPc-DPA-32(NH_2CH_3)	0.142	0.012
$Cd_{33}Se_{33}/Zn_{93}S_{93}$-2(ZnPc)	0.083	0.023

10 Additional nanocrystal systems

10.1 Spectrum of $Cd_{33}Se_{33}/Zn_{93}S_{93}$-2(ZnPc)-DPA

Supplementary Figure 20 shows the $Cd_{33}Se_{33}/Zn_{93}S_{93}$-2(ZnPc)-DPA nanocrystal system. It is nearly identical to the $Cd_{33}Se_{33}/Zn_{93}S_{93}$-2(ZnPc) system in the main manuscript but with an additional DPA molecule. By adding the additional DPA molecule, we observe the occurrence of additional narrow features at around 6.8 eV which are very close in energy to each other, appearing almost as one single broad feature. Even the Fourier transform of the 20,000 time step long-time reference is not able to fully resolve these features. Thus, the $Cd_{33}Se_{33}/Zn_{93}S_{93}$-2(ZnPc)-DPA nanocrystal system is an exceptionally challenging test case with closely spaced narrow features embedded in a large amount of quasi-continuum.

BYND is able to correctly reproduce all spectral features with only 3,000 time steps. While the absolute intensities of the narrow features is overestimated for this signal length, the their relative intensities are almost correctly reproduced.

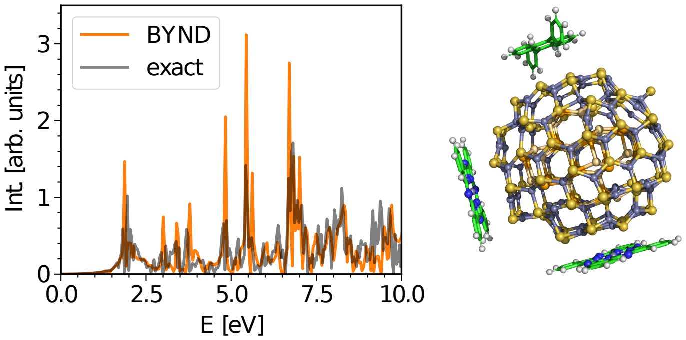

Supplementary Figure 20: Additional $Cd_{33}Se_{33}/Zn_{93}S_{93}$-2(ZnPc)-DPA nanocrystal models which we use in order to estimate spectral convergence. The shown spectrum was obtained for a signal length of 3,000 time steps.

10.2 For computing time estimation

For our computing time estimation in the main text, we use realistic quantum dot models. At this size, the RT-TDDFT calculation dominates the total cost of BYND by far. Therefore, the total computing time for the BYND approach is computed from the FHIaims total run-time estimate of RT-TDDFT, which we divided by a factor of 11 for the 1,500 time step case and which we divided by 5.5 for the 3,000 time step case. As we show in the main text, a speed up of a factor of 11 is a reasonable assumption. In Supplementary Figure 21 we display the nanocrystal models used for this estimate.

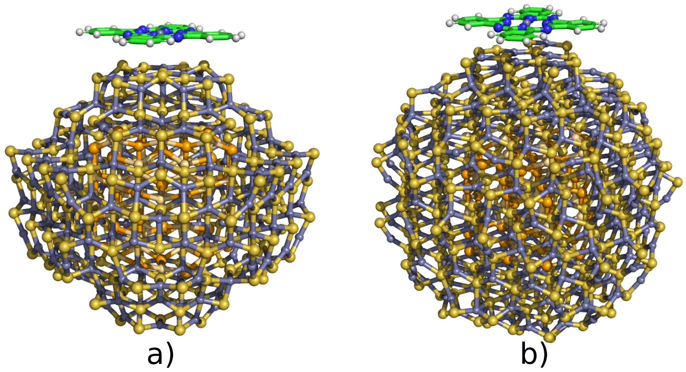

Supplementary Figure 21: Additional nanocrystal models which we use in order to estimate the required computing time. a) $Cd_{48}Se_{48}/Zn_{213}S_{213}$-ZnPc and b) $Cd_{48}Se_{48}/Zn_{327}S_{327}$-ZnPc

Supplementary References

1. Curtis, S. The classification of greedy algorithms. *Sci. Comput. Program.* **49,** 125–157. ISSN: 0167-6423 (2003).
2. Andrade, X., Sanders, J. N. & Aspuru-Guzik, A. Application of compressed sensing to the simulation of atomic systems. *Proc. Natl. Acad. Sci. U.S.A.* **109,** 13928–13933 (2012).
3. Mahdaoui, A. E., Ouahabi, A. & Moulay, M. S. Image denoising using a compressive sensing approach based on regularization constraints. *Sensors* **22.** ISSN: 1424-8220 (2022).
4. Orović, I., Papić, V., Ioana, C., Li, X. & Stanković, S. Compressive sensing in signal processing: Algorithms and transform domain formulations. *Math. Probl. Eng.* **2016,** 7616393. ISSN: 1024-123X (Oct. 2016).
5. Hoerl, A. E. & Kennard, R. W. Ridge regression: Biased estimation for nonorthogonal problems. *Technometrics* **12,** 55–67 (1970).
6. Tikhonov, A. N. Solution of incorrectly formulated problems and the regularization method. *Soviet Math. Dokl.* **4,** 1035–1038 (1963).
7. Blum, V. *et al.* Ab initio molecular simulations with numeric atom-centered orbitals. *Comput. Phys. Commun.* **180,** 2175–2196. ISSN: 0010-4655 (2009).
8. Perdew, J. P., Burke, K. & Ernzerhof, M. Generalized gradient approximation made simple. *Phys. Rev. Lett.* **77,** 3865–3868 (18 Oct. 1996).
9. Hekele, J., Yao, Y., Kanai, Y., Blum, V. & Kratzer, P. All-electron real-time and imaginary-time time-dependent density functional theory within a numeric atom-centered basis function framework. *J. Chem. Phys.* **155,** 154801. ISSN: 0021-9606 (Oct. 2021).
10. Vosko, S. H., Wilk, L. & Nusair, M. Accurate spin-dependent electron liquid correlation energies for local spin density calculations: a critical analysis. *Can. J. Phys.* **58,** 1200–1211 (1980).
11. Adamo, C. & Barone, V. Toward reliable density functional methods without adjustable parameters: The PBE0 model. *J. Chem. Phys.* **110,** 6158–6170. ISSN: 0021-9606 (Apr. 1999).
12. Ernzerhof, M. & Scuseria, G. E. Assessment of the Perdew–Burke–Ernzerhof exchange-correlation functional. *J. Chem. Phys.* **110,** 5029–5036. ISSN: 0021-9606 (Mar. 1999).
13. George Jr, A. *et al. Essentials of Padé approximants* (Elsevier, 1975).
14. Bruner, A., LaMaster, D. & Lopata, K. Accelerated broadband spectra using transition dipole decomposition and Padé approximants. *J. Chem. Theory Comput.* **12.** PMID: 27359347, 3741–3750 (2016).
