# Supplementary material for: Super-resolution techniques to simulate electronic spectra of large molecular systems
Source: Nat Commun. 2024 Sep 12;15:8001. doi: 10.1038/s41467-024-52368-5 (PMC11393058; doi:10.1038/s41467-024-52368-5)
Supplement: Supplementary file 3 — Description of Additional Supplementary Files [file 41467_2024_52368_MOESM3_ESM.pdf]

Description of Additional Supplementary Files:

Supplementary Data 1:

sma_frq:

benzene.txt

Initial excitation energies for Benzene.

butadine.txt

Initial excitation energies for Butadine.

cd33_znpc_znpc.txt

Initial excitation energies for Cd33Se33/Zn93S93-

2(ZnPc).

cd33_znpc_znpc_dpa.txt

Initial excitation energies for Cd33Se33/Zn93S93-

2(ZnPc)-DPA.

f-cororene.txt

Initial excitation energies for F-cororene.

nano_tube.txt

Initial excitation energies for Zinc-porphyrin

molecules on a carbon nanotube.

p3b2.txt

Initial excitation energies for P3b2.

qd_znpc.txt

Initial excitation energies for Cd38Se38-ZnPc32(NH2CH3).

qd_znpc_dpa.txt

Initial excitation energies for Cd38Se38-ZnPc-DPA32(NH2CH3).

ru_tio2.txt

Initial excitation energies for Cis-[Ru(4,4’-COOH-2,2’-

bpy)2(NCS)2] on an anatase (101).

znpc_aggregate.txt

Initial excitation energies for Molecular ZnPc j-aggregate.

znpc_si.txt

Initial excitation energies for ZnPc moleculs on Si

(111).
